# High‐resolution deuterium metabolic imaging of the human brain at 9.4 T using phase‐cycled balanced SSFP spectral–spatial acquisitions

**DOI:** 10.1002/mrm.70114

**Published:** 2025-10-13

**Authors:** Praveen Iyyappan Valsala, Rolf Pohmann, Rahel Heule, Georgiy A. Solomakha, Nikolai I. Avdievich, Jörn Engelmann, Laura Kuebler, André F. Martins, Klaus Scheffler

**Affiliations:** ^1^ High Field Magnetic Resonance, Max‐Planck Institute for Biological Cybernetics Tübingen Germany; ^2^ Department of Biomedical Magnetic Resonance Eberhard Karls University Tübingen Tübingen Germany; ^3^ Center for MR Research, University Children's Hospital Zurich Switzerland; ^4^ Werner Siemens Imaging Center, Department for Preclinical Imaging and Radiopharmacy Eberhard Karls University Tübingen Tübingen Germany; ^5^ Cluster of Excellence iFIT (EXC 2180) “Image‐Guided and Functionally Instructed Tumor Therapies”, Eberhard Karls University Tübingen Tübingen Germany; ^6^ German Cancer Consortium (DKTK), partner site Tübingen, German Cancer Research Center (DKFZ) Heidelberg Germany

**Keywords:** bSSFP, deuterium metabolic imaging, IDEAL, SNR, ultra‐high‐field

## Abstract

**Purpose:**

The aim was to improve the sensitivity and robustness against B_0_ inhomogeneities of deuterium metabolic imaging (DMI) using phase‐cycled balanced SSFP (bSSFP) methods at 9.4 T.

**Methods:**

We investigated two variants of phase‐cycled bSSFP acquisitions, namely uniformly weighted multi‐echo and acquisition‐weighted chemical shift imaging (CSI) to improve the SNR of DMI in the brain after oral [6,6′‐^2^H_2_]‐glucose intake. Phase‐cycling was introduced to reduce the off‐resonance sensitivity of bSSFP, incurring a moderate SNR loss. Two SNR optimal methods for obtaining metabolite amplitudes from the phase‐cycled bSSFP data were proposed. The SNR performance of the two bSSFP variants was compared with the SNR‐optimized vendor's standard CSI. Additionally, in vivo T_1_ and T_2_ of deuterium metabolites were estimated.

**Results:**

High‐resolution whole‐brain dynamic DMI maps were obtained for all acquisitions. The CSI variant of phase‐cycled bSSFP achieved an average SNR increase of 16% and 25% for glucose and glutamate + glutamine (Glx), respectively, compared to the SNR‐optimized vendor's standard CSI. Phase‐cycling improved the bSSFP metabolite estimation and provided additional spectral encoding at the cost of a 10% to 20% SNR loss. Compared to the CSI variant of bSSFP acquisition, the multi‐echo variant exhibited up to 35% lower SNR performance because of uniform k‐space weighting and less efficient readout. However, it achieved higher resolutions than acquisition‐weighted CSI protocols and showed several qualitative improvements.

**Conclusion:**

We demonstrated the feasibility of using two phase‐cycled bSSFP acquisitions for off‐resonance insensitive high‐resolution [6,6′‐^2^H_2_]‐glucose DMI studies in the human brain. bSSFP acquisitions have potential to improve the sensitivity of DMI despite the SNR loss of phase‐cycling and other human scanner constraints.

## 1 INTRODUCTION

Deuterium metabolic imaging (DMI) has recently gained traction in the field of metabolic research for the study of normal and diseased metabolic pathways in pathologies such as cancer, diabetes, and neurodegenerative diseases.^1^, ^2^ Because of the low natural abundance of deuterium in the human body, metabolic studies are performed with deuterated tracers. Most studies use [6,6′‐^2^H_2_]‐labeled glucose, which provides comprehensive information on glucose uptake, glycolysis and the tricarboxylic acid (TCA) cycle. In contrast, the routinely used fluorodeoxyglucose (FDG) positron emission tomography (PET)^3^ in clinical practice only provides information on glucose uptake. Furthermore, the low natural abundance and the few resonances of the exogenous tracer and its downstream metabolites enable straightforward acquisition and dynamic metabolite concentration estimation without many confounders.^4^ This offers an advantage over other experimental MR metabolic imaging methods, including ^1^H MRSI,^5^ ^13^C MRSI,^6^ and glycoCEST.^7^ The non‐radioactive deuterium tracers are also suitable for longitudinal studies because they can be safely taken repeatedly.

Despite its promises, the low sensitivity of DMI resulting from the low gyromagnetic ratio and low concentration of deuterated metabolites limits its practical applicability. DMI studies are predominantly conducted at high field strengths because the SNR increases supra‐linearly with the static magnetic field^8^ ($\mathrm{SNR}\propto B_{0}^{1.7}$). Glucose‐DMI investigations in the human brain have reported spatio‐temporal resolutions ranging from 3 mL in 10 min at 9.4 T to 14 mL in 39 min at 3 T^2^.^9^, ^10^, ^11^, ^12^ The acquisition weighting used in all these studies to improve SNR further degrades the nominal resolution by a factor of 4 to 8. Therefore, improving the sensitivity of DMI is highly desirable. Recently, balanced SSFP (bSSFP) acquisition^13^ rather than RF‐spoiled gradient‐echo (also referred to as FLASH) acquisition has been proposed in 15.2 T pre‐clinical DMI studies.^14^, ^15^ The rationale behind this was the much higher T_2_/T_1_ ratio of deuterated metabolites compared to protons, even at high field strengths, yielding considerable signal enhancement when using bSSFP acquisitions.^16^ However, these benefits are only realized when the region and metabolite of interest fall within the passband of the bSSFP frequency response. Because of the significant B_0_ inhomogeneity in the human head, bSSFP acquisitions can be repeated at multiple linear RF phase increments to mitigate off‐resonance banding artifacts and improve coverage, referred to as phase cycles.^13^ However, this can reduce SNR by up to 40%, depending on the combination method used.^17^, ^18^

In this clinical translational study, the feasibility of using phase‐cycled bSSFP acquisitions to improve [6,6′‐^2^H_2_]‐glucose DMI in the healthy human brain at 9.4 T is investigated. To this end, phase‐cycled bSSFP sequences using two popular 3D encoding schemes, namely chemical shift imaging (CSI) and spectroscopic multi‐echo (ME), were compared. Two spectral fitting methods were developed for the phase‐cycled bSSFP data to obtain reliable and SNR optimal metabolite maps. The SNR performance and spectral–spatial encoding of these two phase‐cycled bSSFP acquisition schemes were compared to the vendor's standard 3D CSI sequence. The DMI protocols were designed to maximize SNR based on the simulation of the acquired signal using the measured in vivo T_1_, T_2_, and T_2_* relaxation times. The spectral separation of the designed protocols was verified using a phantom containing all four (water, glucose, glutamic acid, and lactate) deuterated compounds detected in in vivo [6,6′‐^2^H_2_]‐glucose DMI.^4^

## 2 METHODS

### 2.1 DMI sequences

In this study, three MR sequences were used for DMI measurements, namely: the vendor's standard 3D CSI as the reference method and two new phase‐cycled bSSFP variants with CSI and ME encoding schemes referred to as CSI‐PC‐bSSFP and ME‐PC‐bSSFP, respectively, throughout this work. Both bSSFP sequences have a configurable preparation module with a flip angle ramp at the beginning and dummy cycles in between the phase cycles to reach steady‐state. The vendor's standard CSI sequence uses a gradient‐spoiled acquisition without RF‐ spoiling,^19^ commonly referred to as FISP/SSFP‐FID in the literature.^20^

### 2.2 Simulation

SNR for different metabolites were simulated for the three sequences with the analytical steady‐state signal equations of FISP^21^ and bSSFP^22^ acquisition. The measured in vivo relaxation times (T_1_, T_2_, and T_2_*) of the metabolites and system constraints (energy deposition limit and B_0_ inhomogeneity) were included to estimate the signal dependence as realistic as possible. The effects of T_2_* decay during signal readout, ADC (analog‐to‐digital converter) duty cycle and phase‐cycling during bSSFP acquisition were modeled into the signal equation. In addition, the minimum echo spacing/readout duration for reliable spectral separation was determined as that required to maximize effective number of signal averages (see Figure S1).^23^

### 2.3 Measurement setup

All measurements were performed on a 9.4 T Siemens Magnetom whole‐body human scanner. A double‐tuned phased array RF coil with 8 TxRx/2Rx deuterium channels and 10 TxRx proton channels^9^, ^24^ was used. The new software baseline (VE12U) of the scanner natively supports deuterium imaging with an additional license.

### 2.4 Phantom

To investigate the SNR improvements and spectral resolution of different acquisition methods, we constructed an 18 cm diameter spherical phantom with nine 20 mL vials containing different metabolite mixtures (deuterated water, [6,6′‐^2^H_2_]‐glucose, [4,4′‐^2^H_2_]‐glutamic acid hydrochloride and sodium [3,3,3‐^2^H_3_]‐lactate). The vials were distributed along the outer periphery to minimize bias because of transmit and receive field inhomogeneity. A detailed description of the phantom construction is provided in the shared data repository.

### 2.5 Human subjects

Five healthy subjects (age = 32 ± 5 years, 2 males) took part in the study and were asked to fast overnight and ingest [6,6′‐^2^H_2_]‐glucose solution (0.75 g/kg body weight) before the measurements. All in vivo experiments were approved by our local ethics committee and were only performed after obtaining written consent from the study participants. The final optimized protocols were tested on three subjects, while the data from two additional subjects was used for non‐localized relaxometry and protocol optimization. The measurements were conducted over a period of approximately 2 h, during which all three DMI protocols described below were interleaved at least twice. In addition, non‐localized relaxometry and structural scans were performed.

### 2.6 DMI protocols

All in vivo DMI protocols were designed for 10 min of acquisition time irrespective of the resolution and other acquisition parameters to facilitate comparison. Acquisition parameters of all spectral–spatial ^2^H imaging sequences are summarized in Table 1. All protocols used non‐selective rectangular RF pulses to maximize ADC duty cycle and stay within specific absorption rate (SAR) restrictions. The readout length of 14.9 ms for CSI‐PC‐bSSFP as well as 5 echoes with an echo spacing of 3.4 ms for ME‐PC‐bSSFP was sufficient to achieve spectral separation of all four deuterated metabolites at 9.4 T (see Figure S1). CSI‐PC‐bSSFP and ME‐PC‐bSSFP use 4 and 18 phase cycles, respectively, to reduce off‐resonance sensitivity. Mono‐polar readout gradients were used in ME‐PC‐bSSFP because of the low bandwidth readout to ensure unidirectional pixel shifts. In the case of the standard CSI protocol, an SNR‐optimal readout length of 1.26 times T_2_* (assuming a T_2_* of 22 ms) was used, which is longer than the minimum readout length required for spectral separation and balances T_1_ regrowth with T_2_* decay to provide optimal SNR, as shown in prior studies.^25^, ^26^ To increase the number of signal averages, both CSI protocols used Hamming acquisition weighting, whereas ME‐PC‐bSSFP used elliptical scanning. The resolution loss because of Hamming acquisition weighting and the elliptical k‐space sampling was estimated from the full width of point spread function (PSF) at 64% peak height. In addition to the standard protocols, an adapted protocol without phase‐cycling was used in a phantom measurement to demonstrate off‐resonance insensitivity and additional spectral encoding provided by phase‐cycling.

**TABLE 1:** Main sequence parameters of all three DMI protocols used in this study.

					RF pulse				
Protocol	FOV (mm)	Matrix size	TR (ms)	Repetitions (averages/ phase cycles)	FA (°)	Dur (ms)	ADC duty cycle (%)	Nominal voxel volume (mL)	PSF voxel volume (mL)
CSI	208 × 208 × 208	25 × 25 × 25	36	12/n.a	41	0.5	79	0.58	4
CSI‐PC‐ bSSFP	208 × 208 × 208	25 × 25 × 25	19	6/4	50	1.4	78	0.58	4.3
ME‐PC‐bSSFP	400 × 200 × 300	32 × 16 × 24	19	5/18	50	1.4	71	1.95	2.67

Abbreviations: ADC, analog to digital converter; bSSFP, balanced SSFP; CSI, chemical shift imaging; CSI‐PC‐bSSFP, CSI phase‐cycled balanced SSFP; Dur, RF pulse duration; FA, flip angle; ME‐PC‐bSSFP, multi‐echo phase‐cycled bSSFP; PSF, point spread function; n.a, not available.

### 2.7 Image reconstruction

The acquired data was noise‐prewhitened, averaged, zero‐padded to double the encoding matrix size and reconstructed using a 3D fast Fourier transform (FFT) along the three physical dimensions to obtain the reconstructed volume at different time points and phase cycles. After reconstruction, adaptive coil combination^27^ was used for both CSI and multi‐echo data to combine multi‐channel images. The coil weights were calculated by averaging the image volume across the echo‐time and phase‐cycling dimension.

### 2.8 Spectral fitting

**FIGURE 1:**
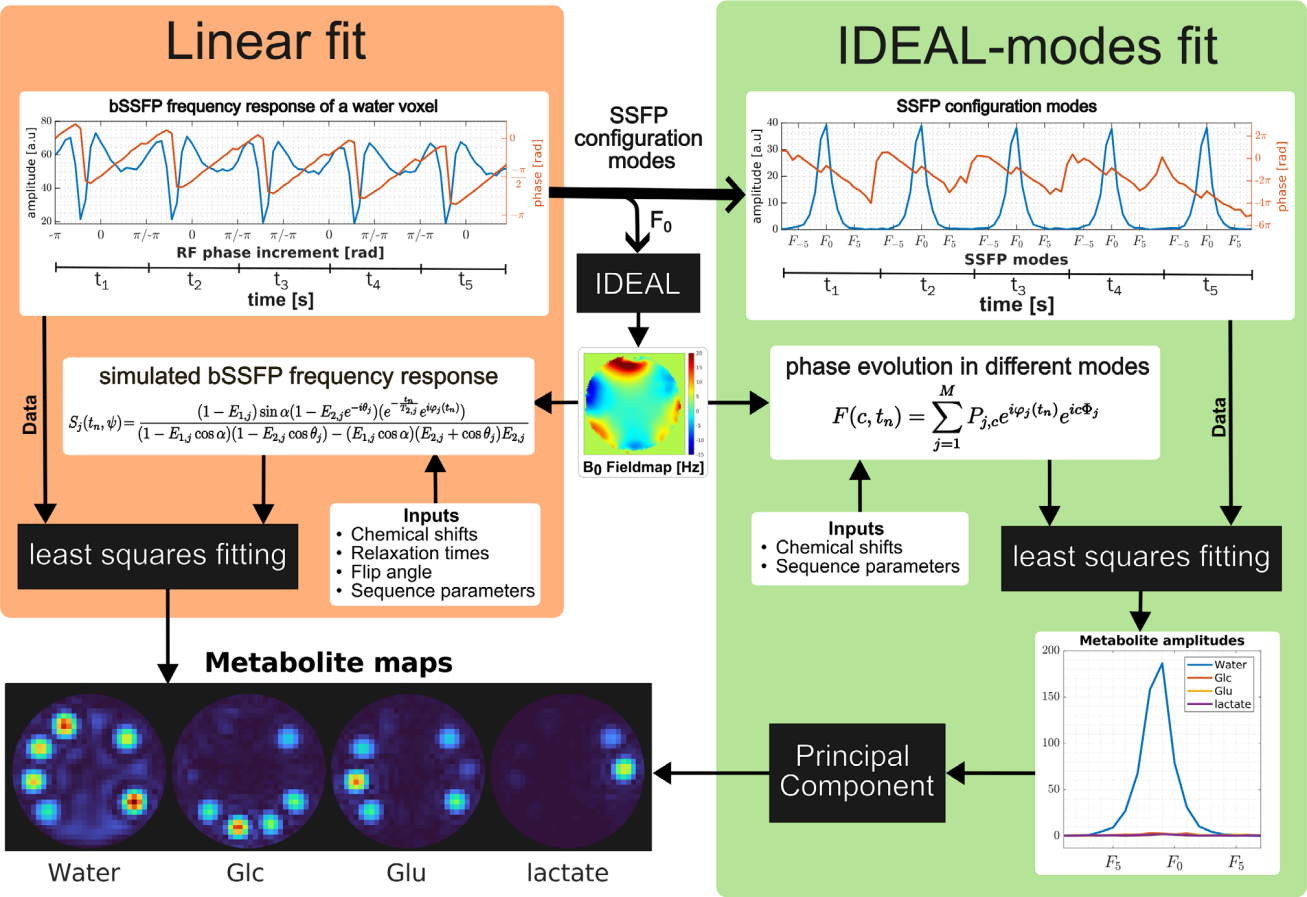
Overview of the two proposed data processing methods for phase‐cycled balanced SSFP (bSSFP) data. An exemplary bSSFP frequency response of a water voxel from a multi‐echo phase‐cycled bSSFP (ME‐PC‐bSSFP) phantom experiment for all TEs is fitted to the simulated bSSFP frequency response using the linear fit method. For the IDEAL‐modes method, only the phase evolution is fitted to the individual SSFP configuration modes and the metabolite amplitudes across different SSFP modes are optimally combined to obtain final metabolite maps. Both methods rely on the B_0_ field map estimated from complex‐averaged phase‐cycled bSSFP data (lowest‐order SSFP‐FID/F_0_ mode) at multiple TEs obtained by the IDEAL algorithm.

The metabolite amplitudes from the standard CSI datasets were estimated by simultaneously fitting the phase evolution of the metabolites along the readout and B_0_ off‐resonance dimension using the standard IDEAL algorithm.^28^ In the case of phase‐cycled bSSFP data, we propose two novel methods to handle the additional amplitude and phase modulations introduced by the phase‐cycling: IDEAL‐modes fit and linear fit. The key steps involved in those two proposed methods are schematically illustrated in Figure 1. The IDEAL‐modes fit used a relatively simple phase evolution signal model similar to the standard IDEAL algorithm in the SSFP configuration space^13^ followed by a data‐driven approach for optimally combining metabolite amplitudes across SSFP modes to maximize SNR. In the linear fit, phase‐cycling related amplitude and phase modulations in the data were explicitly formulated in the signal model to provide additional information for spectral separation. The complex averaged phase‐cycled data without bandings (F_0_ mode)^29^ were used to estimate the B_0_ off‐resonance map with the conventional IDEAL algorithm.^28^

In the IDEAL‐modes fit, SSFP configuration modes were estimated via a discrete Fourier transform (DFT) of the acquired phase cycles to remove the off‐resonance‐dependent spatial amplitude variations (i.e., bandings). As a result of the finite sampling of the bSSFP frequency response, the highest SSFP configuration order C that can be estimated with K phase cycles is given by *C* = *K*/2 – 1. Extending equation (10) from Nyugen and Bieri^29^ for M metabolites, the SSFP mode amplitude $F\left(c,t_{n}\right)$ of an image pixel at a discrete time point $t_{n}$ (*n* = 1, …, *N*) and configuration order **c** (*c* = −*C*, …, 0, …, *C*), ignoring T_2_ relaxation effects, is given by

(1) $$F\left(c,t_{n}\right)=\sum_{j=1}^{M}P_{j,c}e^{i\varphi_{j}\left(t_{n}\right)}e^{\mathrm{ic}\Phi_{j}},$$

where $P_{j,c}$ is the *j*th metabolite amplitude for SSFP configuration order **c**. The phase terms $\varphi_{j}\left(t_{n}\right)≔\left(t_{n}/\mathrm{TR}\right)\Phi_{j}$ and $\Phi_{j}≔2\pi \left(\Delta f_{j}+\Delta f_{0}\right)\mathrm{TR}$ refer to the phase accumulated by the *j*th metabolite at a discrete TE $t_{n}$ and the phase accumulated during each TR, respectively. The off‐resonance terms $\Delta f_{j}$ and $\Delta f_{0}$ refer to the chemical shift of the *j*th metabolite and the B_0_‐related off‐resonance in Hz at the spatial position, respectively. The intermediate metabolite amplitudes $P_{j,c}$ of all the modes, estimated via the pseudo‐inverse of Eq. ([1]), were optimally combined with the principal eigenvector. A detailed description of the computational steps involved in the IDEAL‐modes algorithm is provided in Figure S2. Because of the flexibility of this method and slightly better SNR performance than the linear fit method, it was used throughout this work unless specified otherwise.

In the linear fit, the acquired phase‐cycled bSSFP data were fitted to bSSFP frequency responses, simulated with a more complete signal model^22^ than the simple phase evolution model used in the IDEAL‐modes approach, using a linear least squares algorithm to estimate the metabolite amplitudes directly. The bSSFP signal $S_{j}$ of the *j*th metabolite with chemical shift $\Delta f_{j}$ at discrete time point $t_{n}$ (*n* = 1, …, *N*) was calculated by adding phase evolution and relaxation terms during free induction decay to the transverse magnetization after excitation as described by Ganter in equations (42–44).^30^ In addition to the chemical shift $\Delta f_{j}$ and time point $t_{n}$, the signal depends on off‐resonance $\Delta f_{0}$, T_1_, and T_2_ relaxation times, as well as acquisition parameters (flip angle α, RF phase increment ψ, repetition time TR):

(2) $$S_{j}\left(t_{n},\psi \right)=\frac{\left(1-E_{1,j}\right)\sin\alpha \left(1-E_{2,j}e^{-i\theta_{j}}\right)\left(e^{-\frac{t_{n}}{T_{2,j}}}e^{i\varphi_{j}\left(t_{n}\right)}\right)}{\begin{array}{l} \left(1-E_{1,j}\cos\alpha \right)\left(1-E_{2,j}\cos\theta_{j}\right)\\ -\left(E_{1,j}-\cos\alpha \right)\left(E_{2,j}-\cos\theta_{j}\right)E_{2,j} \end{array}},$$

with $E_{1,j}≔\exp\left(-\frac{\mathrm{TR}}{T_{1,j}}\right)$ and $E_{2,j}≔\exp\left(-\frac{\mathrm{TR}}{T_{2,j}}\right)$ referring to the relaxation terms of the *j*th metabolite, and $\theta_{j}≔\Phi_{j}-\psi$ denoting the phase difference term.

### 2.9 SNR calculation

For accurate SNR estimation, reconstruction was performed in SNR units^31^ by using appropriate scaling factors during averaging, Fourier transform, and coil combination after noise decorrelation. Additional correction for the anti‐aliasing filter was not necessary because readout oversampling was removed from both noise and image data. The coil normalization^32^ weights estimated during the coil combination step to correct for inhomogeneous receive sensitivity was not applied during SNR analysis. The Euclidean norm of the complex linear combination coefficients of the individual metabolites during spectral separation was used to scale the resulting metabolite maps to SNR units, respectively. Additionally, the SNR maps were normalized with the estimated PSF voxel volume for fair comparison.

### 2.10 Metabolite quantification

In vivo quantitative glucose and glutamate + glutamine (Glx) metabolite maps were obtained using the assumed homogenous ^2^H‐water reference of 10.12 mM throughout the brain^2^, ^33^ as shown by Peters et al.^14^ The theoretical signal models referenced in the section Simulation were used for calibrating the metabolite amplitude ratios. Additionally, 33% label loss in Glx transformation during oxidative metabolism was considered.^34^ In case of multi‐echo acquisitions, the voxel shifts along frequency encoding direction because of chemical shifts during the low‐bandwidth readout was estimated and corrected before metabolite quantification. The calculated voxel shifts were 1.8, 4.8, and 6.8 mm for Glucose, Glx, and lipid/lactate, respectively, if the system frequency is set to the water resonance.

### 2.11 Non‐localized spectroscopy

Non‐localized inversion‐recovery and spin‐echo sequences written in the Pulseq framework^35^ were performed to measure T_1_ and T_2_ relaxation times both in vivo and in vitro. For T_1_ relaxometry, longitudinal relaxation was sampled at 15 TIs ranging from 10 to 1500 ms with a TR of 2.5 s. A 5.2 ms non‐selective adiabatic frequency offset corrected inversion (FOCI) pulse^36^ was used for inversion. In case of T_2_ relaxometry, non‐selective block pulses with durations of 1 and 2 ms were used for excitation and refocusing, respectively, to achieve short TEs. The TEs were distributed between 10 and 700 ms with a TR of 2.5 s. Both T_1_ and T_2_ measurements took 10 min each with 16 averages for each inversion/TE. The measurements were performed approximately 90 min after the labeled glucose intake in case of the in vivo scans.

The relaxometry data was noise decorrelated and coil‐combined using the weighted singular value decomposition (WSVD) method.^37^ To minimize the number of fitted parameters, a combined model incorporating both spectral and temporal (TI or TE) dimensions was used, as previously described by Roig et al.^10^ A non‐linear least squares algorithm was used to simultaneously fit the four metabolites with a three‐parameter Lorentzian model along the spectral axis, and the standard exponential models $S\left(\mathrm{TI}\right)=a\left(1-{\mathrm{be}}^{\left(\frac{-\mathrm{TI}}{T_{1}}\right)}\right)$ and $S\left(\mathrm{TE}\right)=ae^{\left(\frac{-\mathrm{TE}}{T_{2}}\right)}$ for the inversion recovery and spin‐echo data, respectively. A bi‐exponential fit was only required for the in vivo water T_2_ relaxation fit to account for ventricular water.^2^ Metabolite T_2_* relaxation times and chemical shifts were obtained from the linewidth and off‐resonance shift, respectively, of the metabolite peaks in the CSI‐FISP data estimated by the AMARES algorithm.^38^, ^39^ In addition to relaxometry, non‐localized spectra were acquired at 10 different pulse powers to determine the actual flip angle.

## 3 RESULTS

### 3.1 Non‐localized spectroscopy

T_1_ and T_2_ relaxation times estimated from the non‐localized inversion‐recovery and spin‐echo scans are shown in Table 2 for in vivo (*n* = 5) and phantom experiments. The water resonance of the inversion‐recovery data shows slight deviation from the Lorentzian line shape possibly because of the strong B_0_ inhomogeneity at 9.4 T (see Figure S3). The spectral‐fitting quality of inversion‐recovery and spin‐echo data acquired in a representative subject can be found in Figures S3 and S4, respectively. The assumed fraction of the ventricular water compartment with longer T_2_ was estimated to be (18 ± 5)% by bi‐exponential T_2_ relaxation fitting.^2^ The chemical shifts of the ^2^H resonances obtained for the phantom data show an upfield shift of approximately 0.2 ppm with respect to water compared to in vivo.

**TABLE 2:** Relaxation times and chemical shifts of deuterium resonances for in vivo (*n* = 5) and phantom data at 9.4 T.

	Resonances	T_1_ (ms)	T_2_ (ms)	T_2_* (ms)	Chemical shift (ppm)
In vivo	Water	371 ± 28	30 ± 3	21 ± 2	4.70
	Water 2 (18 ± 5%)	n.a	293 ± 34	n.a	n.a
	Glucose	71 ± 5	41 ± 7	14 ± 2	3.80
	Glx	153 ± 4	98 ± 5^a^	23 ± 1	2.35
	Lactate/lipids	139 ± 51	138 ± 29	24 ± 5	1.37
Phantom	Water	488 ± 11	275 ± 1	75 ± 39	4.70
	Glucose	64 ± 8	60 ± 8	22 ± 3	3.62
	Glutamic acid	210 ± 40	122 ± 12	53 ± 14	2.16
	Lactate	266 ± 34	258 ± 13	76 ± 29	1.18

T_1_ and T_2_ relaxation times are derived from non‐localized inversion recovery and spin‐echo acquisitions. Water 2 corresponds to the ventricular water compartment modeled during T_2_ fitting. Median T_2_* and chemical shifts are estimated from the standard CSI data with 0.58 mL voxel volume using the AMARES algorithm.

Abbreviations: CSI, chemical shift imaging; Glx, glutamate + glutamine; n.a, not available.

^a^ The measured T_2_ value is notably higher than previously reported literature values.^2^, ^10^, ^45^

### 3.2 Simulation

**FIGURE 2:**
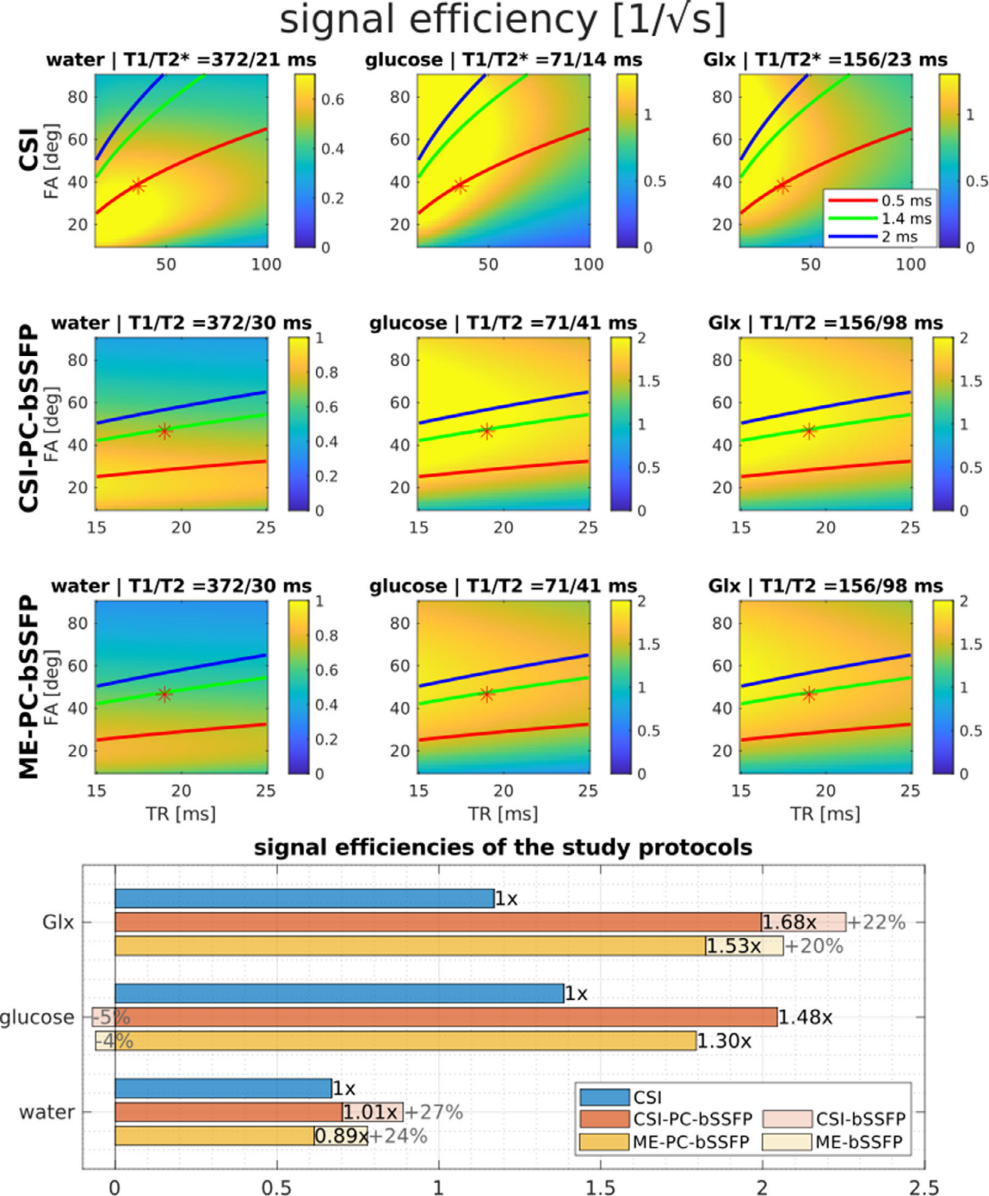
Simulated in vivo signal efficiency ($\text{signal amplitude}/\sqrt{\mathrm{TR}}$) of deuterated metabolites with respect to TR and flip angle for chemical shift imaging (CSI) and two phase‐cycled balanced SSFP (bSSFP) acquisition methods. The measured in vivo relaxation times at 9.4 T used for the simulation are shown in the title of the respective subplots. The specific absorption rate (SAR) limits with respect to TR for three pulse durations are overlaid to show the available parameter space for SNR optimization. The SAR limit is estimated from the time‐averaged RF pulse power and an experimentally determined scaling factor. Note the different TR ranges and color bar scales used to visualize the signal efficiencies of the bSSFP methods as compared to the standard CSI. The asterisks in the plots indicate the parameter combination (flip angle, TR) of the protocols used in this study. The signal efficiencies of the three protocols for water, glucose and Glx are shown in the bottom panel. The relative improvement over the standard CSI is shown on top of the bar plots. The percentage changes in signal efficiency for both bSSFP methods without phase‐cycling (i.e., using only a 180° RF phase increment) are indicated by the transparent bars.

Figure 2 shows the relative signal efficiencies $\left(\frac{s\text{ignal amplitude}}{\surd \mathrm{TR}\;}\right)$ of all DMI protocols in Table 1 for in vivo water, glucose, and Glx relaxation times. According to the simulations, the expected SNR increase of CSI‐PC‐bSSFP is 1%, 48%, and 68% for water, glucose, and Glx, respectively, as compared to the standard CSI acquisition. With the lower ADC duty cycle of ME‐PC‐bSSFP, the respective signal efficiencies decrease by only 9% to 13%. Without phase‐cycling (i.e., simulating only a 180° RF phase increment), a 20% to 27% increase in the signal efficiency of water and Glx would be observed relative to the phase‐cycled bSSFP protocols. The impact of varying the number of phase cycles on the signal efficiency was found to be negligible. A similar simulation for all four metabolites, including lactate, with the relaxation times measured in the phantom is included in Figure S5. For the relatively large T_2_/T_1_ ratios and long T_2_ relaxation times of water and lactate in the phantom, an SNR increase of over two times is predicted while the improvement for glucose and glutamic acid is similar to the in vivo levels.

### 3.3 Phantom studies

**FIGURE 3:**
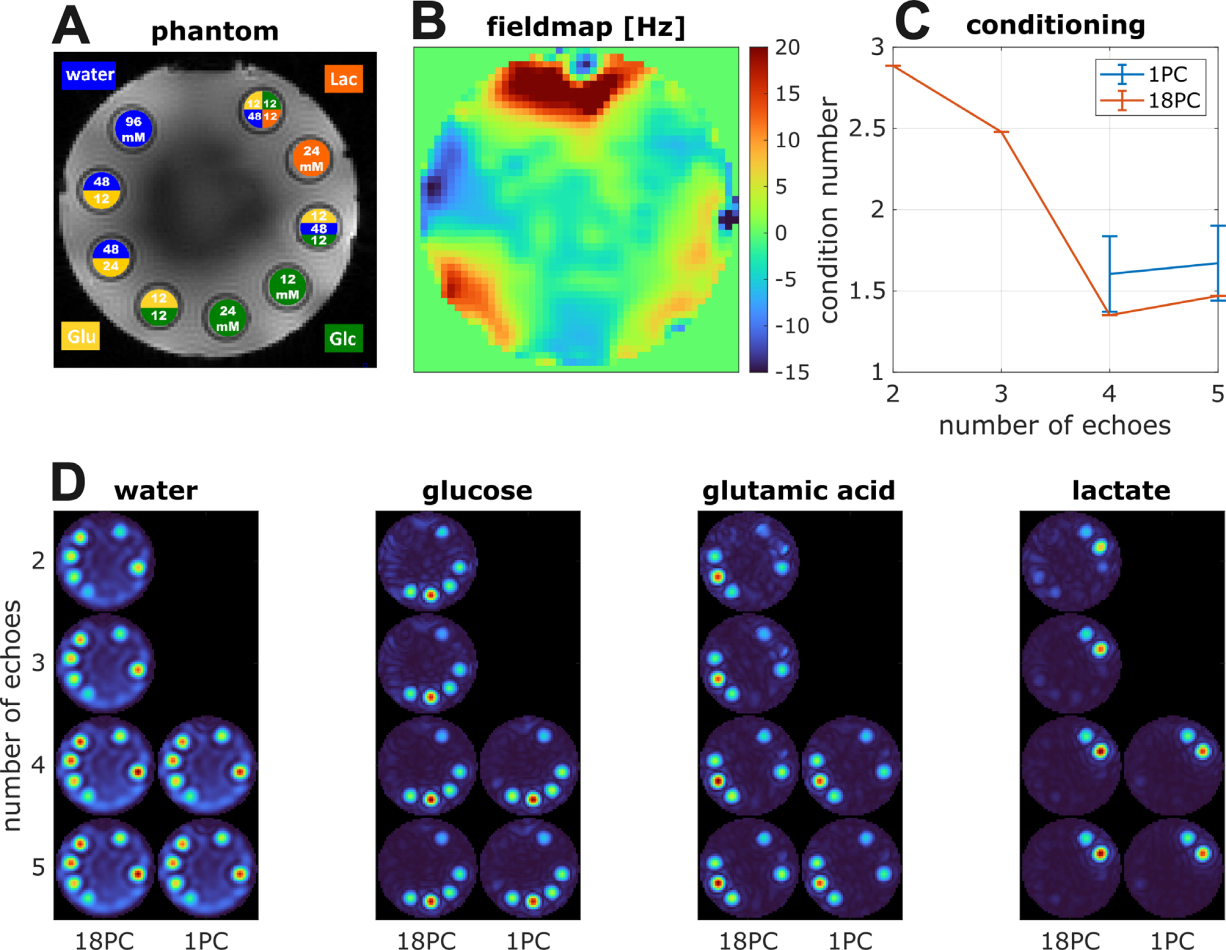
(A) The constructed phantom with various concentrations of four deuterated compounds (blue: Water, green: glucose, yellow: glutamic acid, orange: lactate). (B) Shows the ^2^H B_0_ map estimated using the IDEAL algorithm. (C) Shows better conditioning of the model for matrix inversion with higher number of phase cycles and echoes. The error bars indicate smaller dispersion because of B_0_ off‐resonance with 18 phase cycles. (D) Metabolite maps from the multi‐echo (ME)‐balanced SSFP (bSSFP) protocols with 18 and 1 (RF phase increment of 180°) phase‐cycle/s and different numbers of echoes (echoes were retrospectively removed). The condition number is calculated as the ratio of the largest singular value to the smallest singular value of the design matrix formed with the bSSFP signal model. The error bars indicate dispersion of the condition numbers because of measured inhomogeneities. The four metabolites cannot be separated with less than four echoes in the data without phase‐cycling.

In Figure 3, additional spectral encoding information in the phase cycle dimension is demonstrated by obtaining good spectral separation of all four resonances even with fewer than four echoes when phase‐cycling is performed. The condition number of the system matrix is lower and shows less dispersion because of B_0_ inhomogeneity with more phase cycles. This minimizes additional noise amplification during spectral unmixing. The artifacts because of strong B_0_ inhomogeneity in the water and glucose maps are reduced with more phase cycles. All vials containing the deuterated glutamic acid show an increased water signal possibly because of an additional source of exchangeable deuterium originating from the [4,4′‐^2^H_2_]‐glutamic acid hydrochloride used.

**FIGURE 4:**
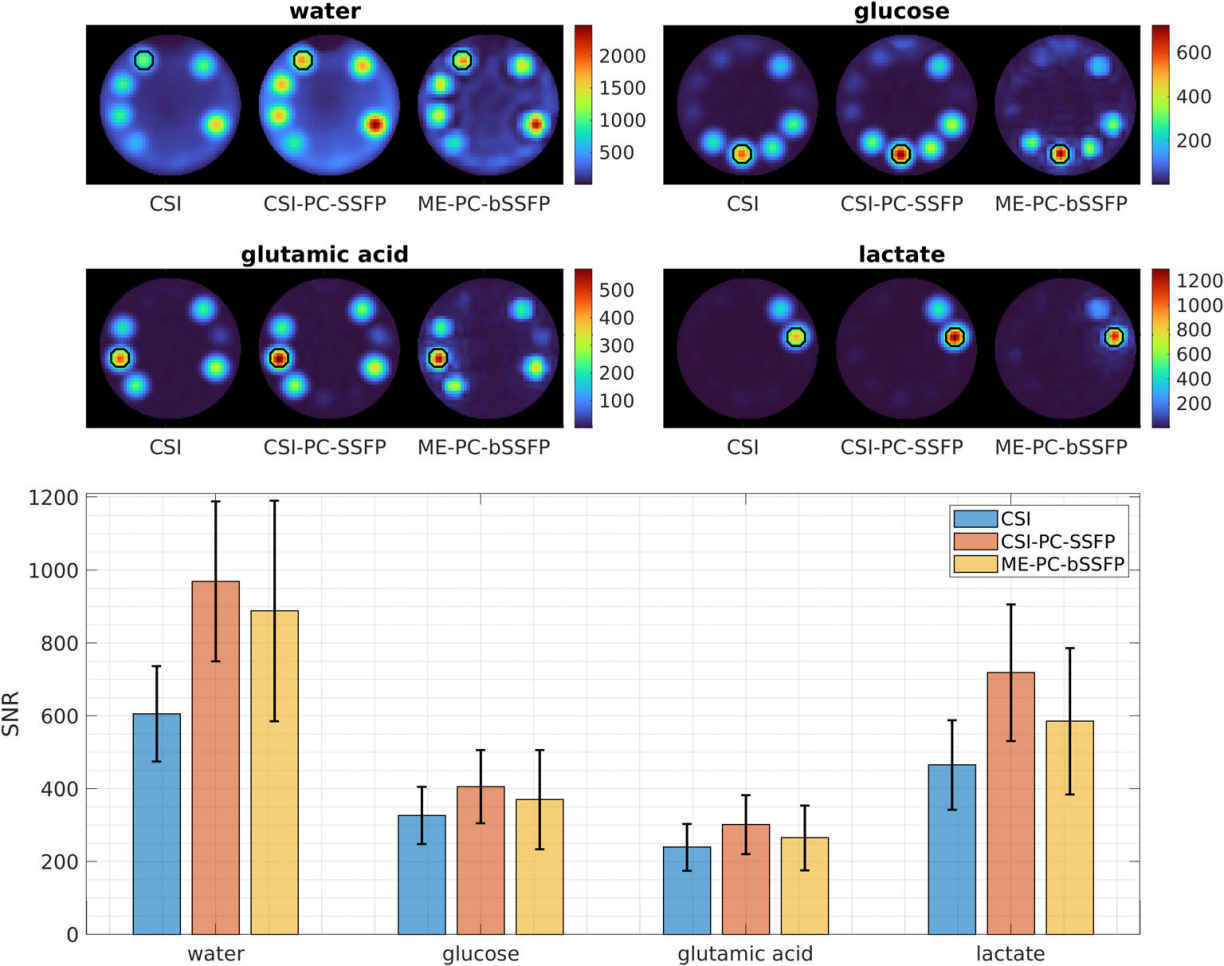
Phantom SNR analysis. The metabolite maps in SNR units obtained using all three investigated protocols are shown for the phantom study. The SNR maps are normalized with the point spread function (PSF) voxel volume of the acquisitions for fair comparison. The normalization factors are 1, 0.94, and 1.52 for standard chemical shift imaging (CSI), CSI phase‐cycled (PC)‐balanced SSFP (bSSFP), and multi‐echo phase‐cycled bSSFP (ME‐PC‐bSSFP), respectively. The bar plots show the mean and SD of the SNR within regions‐of‐interest (ROIs). The vials with the largest concentrations are chosen as ROIs. ROIs are indicated in the metabolite maps with black circles.

The results of the SNR improvement investigation in the phantom for all metabolites using the in vivo protocols are shown in Figure 4. To reduce variability in the small regions of interest (ROIs), four repetitions were combined. The resulting SNR metabolite maps were scaled with the relative PSF voxel volumes from Table 1 for easy comparison. All three protocols exhibit excellent spectral separation of all four metabolites. An improvement in SNR for all four metabolites (i.e., water, glucose, glutamic acid, and lactate, by 60%, 24%, 26% and 54%, respectively) is observed for the CSI‐PC‐bSSFP acquisition as compared to the standard CSI acquisition. However, the ME‐PC‐bSSFP maps reported a lower SNR increase of approximately 47%, 13%, 11%, and 26% for water, glucose, glutamic acid, and lactate, respectively, relative to the standard CSI maps.

### 3.4 In vivo DMI studies

**FIGURE 5:**
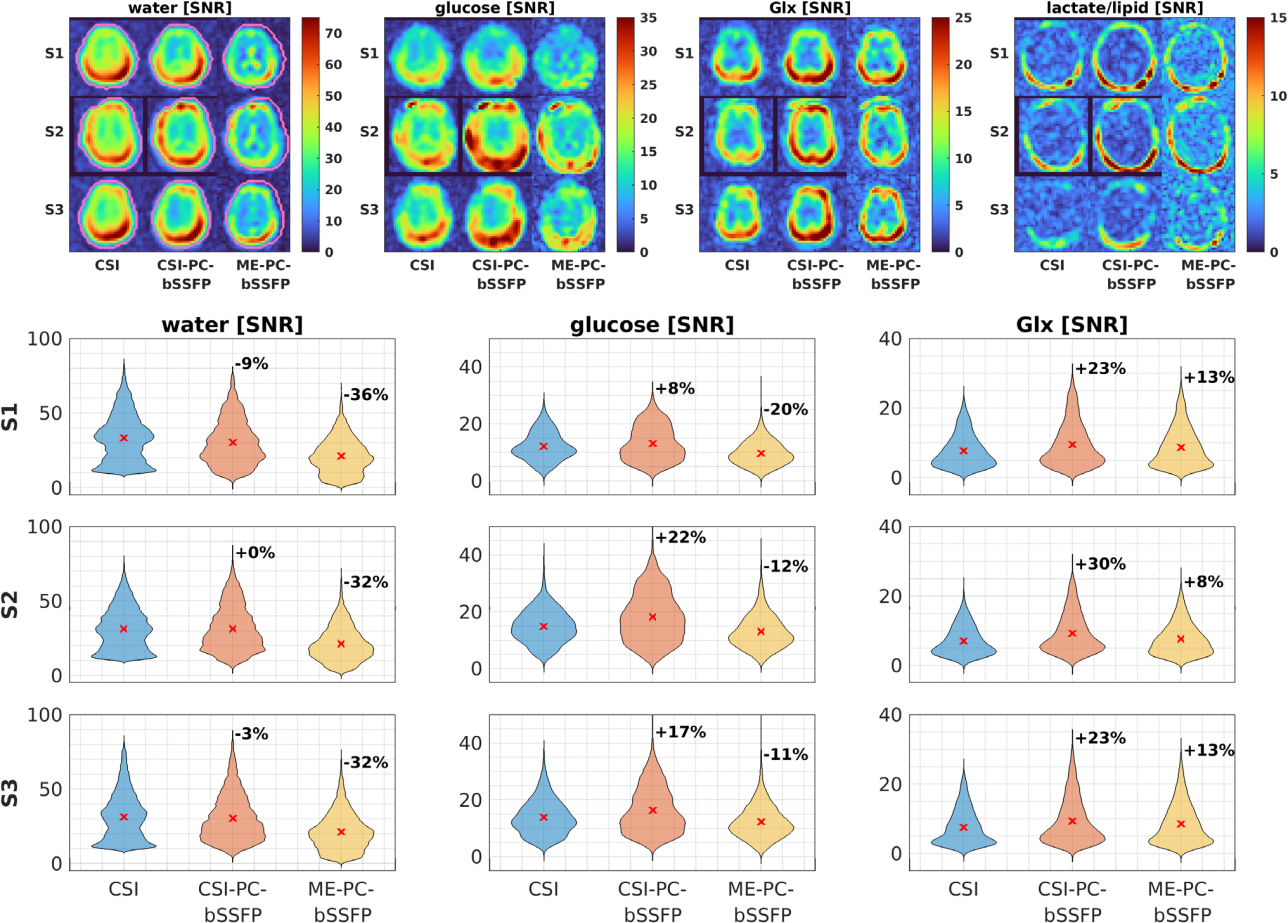
In vivo SNR analysis for all four deuterated metabolites in three subjects. An exemplary axial slice of the metabolite maps (top) and the SNR distribution over the entire brain (bottom) are shown for the three different acquisition methods. The whole‐brain ROIs are depicted as overlaid magenta contour lines in the water SNR image. The average SNR is illustrated as a red cross and the percentage change of the average SNR relative to the standard chemical shift imaging (CSI) acquisition is depicted next to the respective violin plots. The interleaved acquisitions are averaged to minimize the influence of metabolic dynamics. The SNR maps from the CSI phase‐cycled (PC)‐bSSFP and multi‐echo phase‐cycled bSSFP (ME‐PC‐bSSFP) acquisitions are normalized with their corresponding point spread function (PSF) volume relative to the standard CSI, resulting in normalization factors of 0.94 and 1.52, respectively.

Figure 5 shows the overview of in vivo spectral separation and SNR performance of all three acquisition protocols. A representative axial slice of the SNR maps and the whole‐brain SNR distribution of all metabolites are presented for all subjects. The SNR maps from the interleaved DMI scans were averaged according to the acquisition type to minimize the impact of the temporal evolution of metabolites. The SNR maps from CSI‐PC‐bSSFP and ME‐PC‐bSSFP were scaled by a factor of 0.94 and 1.52, respectively, to account for the PSF voxel volume. An average SNR increase of 8% to 22% for glucose and 23% to 30% for Glx is observed for the CSI‐PC‐bSSFP acquisition in comparison to the standard CSI. The water SNR shows only marginal differences between the two acquisitions. ME‐PC‐bSSFP, on the other hand, exhibits an SNR increase of 8% to 13% for Glx, whereas a decrease in SNR by 32% to 36% for water and 11% to 20% for glucose relative to the standard CSI is observed. In subject 2, artifacts in the metabolite maps because of incorrect B_0_ prediction by the IDEAL algorithm in the frontal region are evident.

**FIGURE 6:**
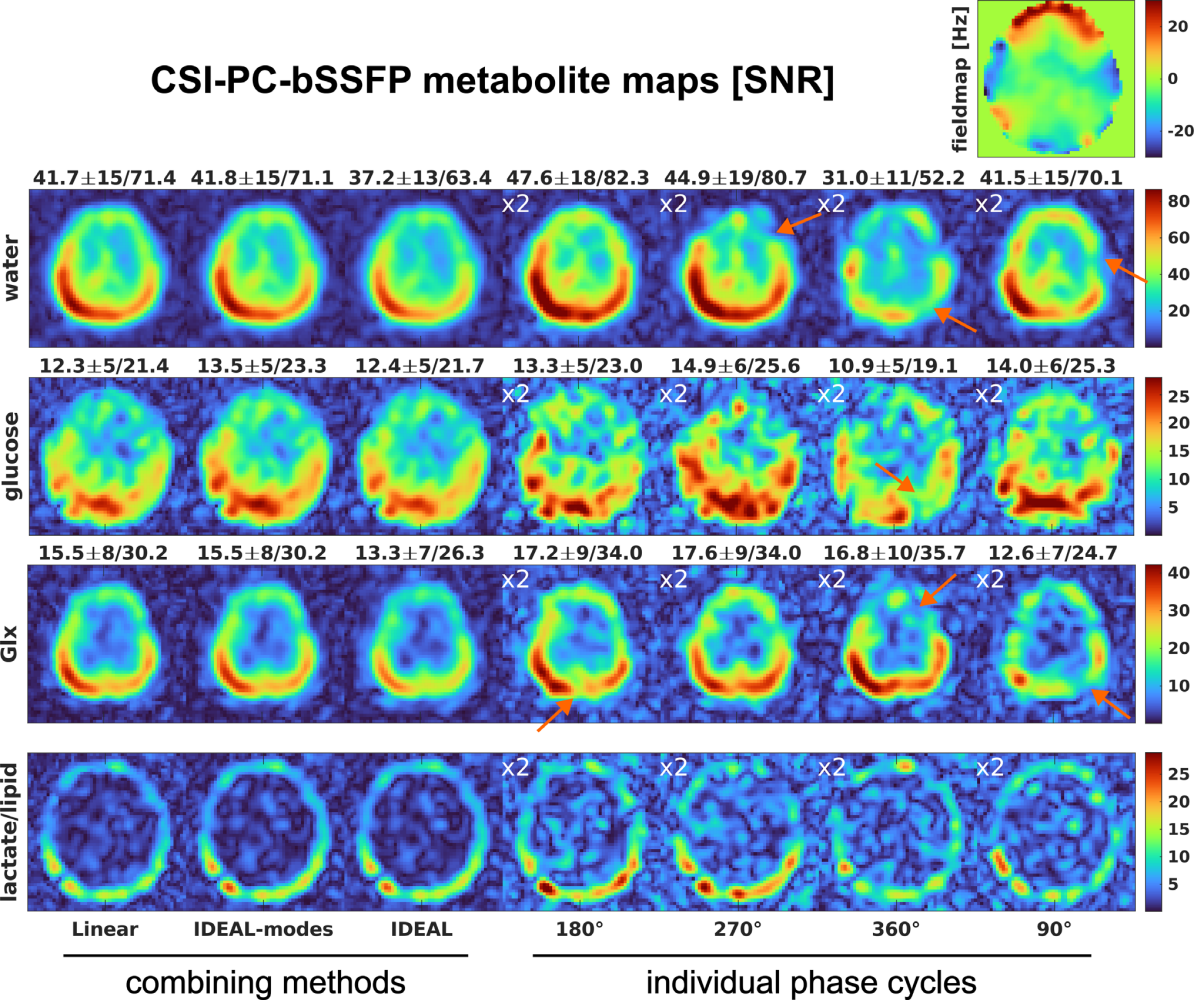
An exemplary transverse slice of the in vivo chemical shift imaging phase‐cycled bSSFP (CSI‐PC‐bSSFP) maps is presented to illustrate the B_0_ insensitivity and SNR loss of phase‐cycling. The four metabolites (water, glucose, glutamate + glutamine [Glx] and lactate/lipid) are displayed along the rows. The first three columns show the three different phase cycle combination methods (linear, IDEAL‐modes, and standard IDEAL algorithm on averaged phase cycles). SNR maps obtained from individual phase cycles (RF phase increments of 180°, 270°, 360°, and 90°) are scaled by a factor of two to account for the short acquisition time and are shown in the last four columns. Shown above the maps are the mean, SD, and 95th percentile (maximum) of the metabolite SNR within the brain mask, formatted as mean ± SD/95^th^ percentile. The orange arrows indicate the incorrect metabolite mapping in the banding regions. The field map in Hz estimated by the IDEAL algorithm is displayed on top right.

In Figure 6, the influence of phase‐cycling and different methods for combining the acquired phase‐cycled data on the SNR is illustrated using the CSI‐PC‐bSSFP data from subject 1. SNR maps obtained from individual phase cycles (RF phase increments of 180°, 270°, 360°, and 90°) and for three different phase cycle combination methods, are investigated. The scaled SNR maps from individual phase cycles show that the measurements without phase‐cycling can achieve higher SNR per unit time at the cost of increased sensitivity to B_0_ inhomogeneity. Furthermore, the optimal RF phase increment for different metabolites is not the same because of the non‐uniform spacing of the chemical shifts. When compared with the scaled individual phase cycle maps, a SNR loss of 20% to 36% depending on the metabolites is observed when metabolite maps are estimated from complex‐averaged data, similar to the 40% loss reported in the literature.^17^ However, the loss of SNR is only 10% to 20% if the proposed linear and IDEAL‐modes methods are used. The IDEAL‐modes had a slightly higher SNR performance than the linear method for glucose.

**FIGURE 7:**
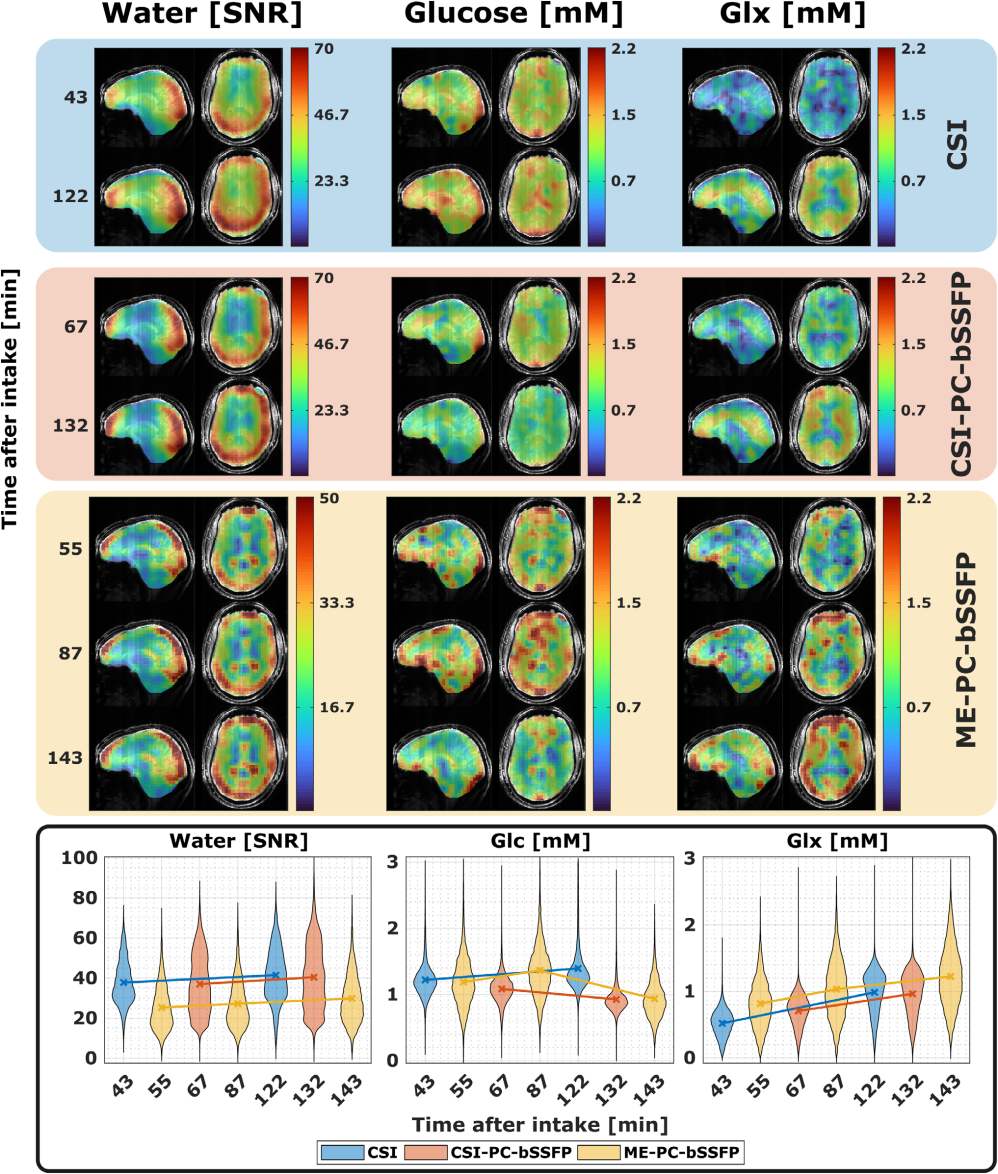
Comparison of quantitative metabolite maps from the standard chemical shift imaging (CSI), CSI phase‐cycled balanced SSFP (CSI‐PC‐bSSFP) and multi‐echo phase‐cycled bSSFP (ME‐PC‐bSSFP) acquisitions in subject 2. The 3D maps of three deuterated metabolites (water in SNR units, quantitative glucose and glutamate + glutamine [Glx] maps in mM) measured at different time points after the glucose intake are overlaid on an anatomical reference. The SNR maps from CSI‐PC‐bSSFP and ME‐PC‐bSSFP were scaled by a factor of 0.94 and 1.52, respectively, to account for the point spread function (PSF) voxel volume for comparison. The violin plots show the time evolution of the ^2^H metabolites across the whole brain. Note the 30 min pause in the dynamic DMI measurements after 87 min used for non‐localized spectroscopy.

The quantitative results of the whole‐brain dynamic DMI measurements performed in subject 2 are summarized in Figure 7. The water SNR maps show strong spatial variations because of the receive sensitivity of the surface coils. The quantitative metabolite maps of glucose and Glx were obtained using the water image acquired at the earliest time point of the corresponding acquisition technique as a reference. The acquired quantitative glucose and Glx maps show a high degree of spatial localization and minimal coil sensitivity bias. In particular, the expected spatial distribution of Glx in the cortical gray matter regions with higher metabolism is visible. The artificially overestimated glucose concentration in the skull because of the lower water concentration in this region is not shown here. The lactate and lipid signals cannot be spectrally separated, which makes it challenging to obtain reliable relaxation times and accurate quantification of these metabolites. The ME‐PC‐bSSFP maps exhibit high spatial granularity compared to the CSI counterparts. Furthermore, ME‐PC‐bSSFP Glx maps consistently predict higher concentrations and show a broader distribution of metabolite concentrations compared to both of the CSI counterparts.

**FIGURE 8:**
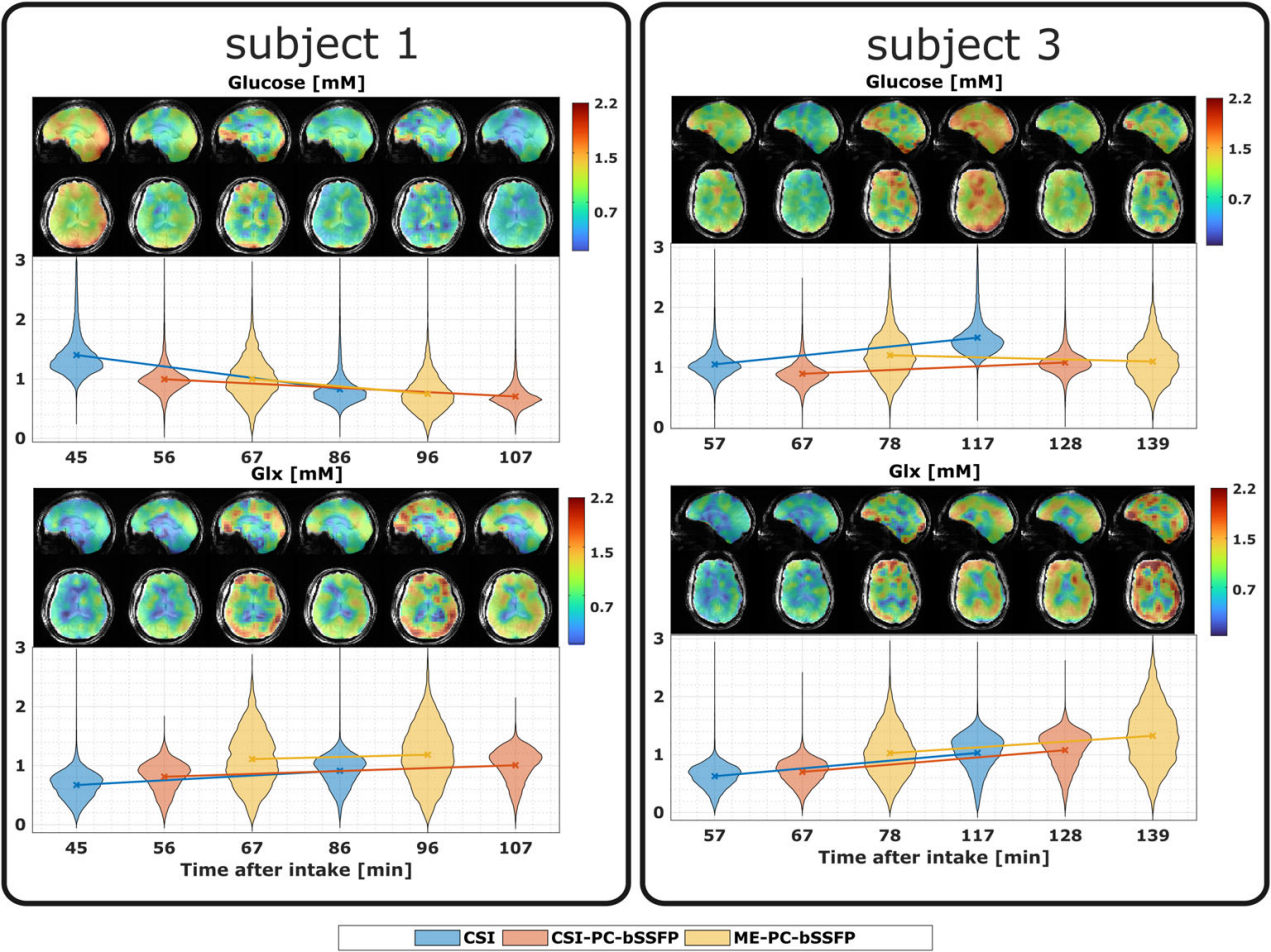
The 3D metabolite quantitative maps of glucose and glutamate + glutamine (Glx) in mM from all the acquisitions in subjects 1 and 3 are overlaid on an anatomical reference. The 3D maps from different time points after the glucose intake are arranged chronologically. The distribution of metabolite concentrations within the brain mask is shown in the corresponding violin plots below. The acquisition method is indicated by the color of the labeled violin plots.

Similarly, the quantitative whole‐brain glucose and Glx maps in mM for all measurements in subjects 1 and 3 are presented in chronological order in Figure 8. The 3D metabolite maps, overlaid over an anatomical reference, are displayed along with the distribution of metabolite concentrations in the brain mask. The temporal evolution of the ^2^H metabolites in the entire brain shows agreement between the different acquisition techniques. However, the time courses of the glucose concentration in the brain of the subjects show significant variability.

## 4 DISCUSSION

In this work, we demonstrated the feasibility of using CSI‐PC‐bSSFP and ME‐PC‐bSSFP acquisitions for off‐resonance insensitive high‐resolution [6,6′‐^2^H_2_]‐labeled glucose DMI studies in the healthy human brain at 9.4 T. The measured in vivo relaxation times of the ^2^H metabolites at 9.4 T were in general agreement with the literature.^2^, ^9^, ^10^, ^40^ The T_1_ values show an increasing trend and the T_2_ values display a moderately decreasing trend with increasing field strength, with the exception of the Glx T_2_ value. The estimated Glx T_2_ value of 98 ms is significantly higher than the reported values (44 ms at 4 T^2^ and 53 ms at 7 T^10^) in the human brain. The intrinsically low Glx signal and the lower SNR of spin‐echo compared with inversion recovery measurements make accurate Glx T_2_ estimation challenging. This potentially higher T_2_ leads to a high signal efficiency estimate during simulation and a higher estimated Glx concentration. Besides that, the ideal Lorentzian line shape assumption during spectral fitting of the non‐localized spectra resulted in a large uncertainty in the fitted peak amplitudes. This translated to large variations across subjects. The bi‐exponential T_1_ relaxation of in vivo water was not apparent in the inversion recovery data. However, the spin‐echo data showed strong bi‐exponential T_2_ relaxation, and therefore, a bi‐exponential model was used to fit the water peak amplitudes.^2^

Using the measured in vivo relaxation times, all in vivo protocols were optimized to improve the signal level of lower concentration metabolites (glucose and Glx) rather than water (see Figure 2). The spectral resolution of all protocols to resolve all ^2^H resonances were verified based on simulations (see Figure S1) and phantom experiments. With the optimized protocols, we achieved a higher nominal resolution in the CSI protocols than the nominal resolution reported in most human DMI studies summarized in Table S1. The PSF analysis suggests that the actually resolved volume with acquisition weighting is approximately 6 to 7 times larger than the nominal voxel volume. In the absence of acquisition weighting, the higher actual resolution of 2.67 mL achieved with ME‐PC‐bSSFP as compared to the CSI variants is clearly visible in the metabolite maps. Because of minor acquisition‐weighting differences (see Figure S6), the actual resolution estimated from the PSF analysis was 4 and 4.3 mL for the standard CSI and CSI‐PC‐bSSFP with a nominal resolution of 0.58 mL, respectively.

CSI‐PC‐bSSFP demonstrated improved average SNR for the detection of glucose (+16%) and Glx (+25%) compared with the SNR‐optimized vendor's standard CSI (see Figure 5). The marginal SNR loss of in vivo water (−4%) can be attributed to the suboptimal flip angle because of the low T_2_/T_1_ ratio. Notably, the 2‐ to 3‐fold SNR gain reported for bSSFP relative to CSI with FLASH acquisition in preclinical studies,^14^, ^15^ was not achieved with CSI‐PC‐bSSFP because of several factors: (1) phase‐cycling for off‐resonance correction resulted in a 10% to 20% in vivo SNR loss; (2) the reference CSI used a short TR to boost SNR at the expense of spectral resolution;^25^, ^26^ (3) the vendor's standard CSI used a non–RF‐spoiled FISP acquisition, which yields higher signal levels than RF‐spoiled FLASH, especially at short TRs;^41^ and (4) human scanner constraints, like peripheral nerve stimulation limits, reducing achievable gradient performance and SAR limit. The signal model used here accurately predicts the higher SNR gains reported in recent bSSFP DMI studies relative to their reference CSI protocols^14^, ^15^, ^42^ (see Figure S7). After accounting for the smaller voxel size, ME‐PC‐bSSFP yielded higher average SNR only for Glx (+11%), whereas the average SNR was lower for water (−33%) and glucose (−14%) compared with the standard CSI (see Figure 5). This represents an average SNR loss of up to 35% in comparison to the similar CSI‐PC‐bSSFP protocol. The simulations predicted an SNR loss of 12% as a result of the lower ADC duty cycle (see Figure 2). The apparent discrepancy can be attributed to the sinc‐shaped PSF, resulting from uniform k‐space weighting with substantial negative lobes (see Figure S6). Theoretically, the negative lobes in the PSF of uniform weighting can lead to a substantial SNR decline, with a potential factor of up to 2.2, depending on the acquisition parameters.^15^, ^43^ In our case, a theoretical SNR reduction by a factor of 1.66 was estimated. This finding largely accounts for the observed underperformance of the ME‐PC‐bSSFP compared to the CSI‐PC‐bSSFP.

In phantom experiments, water and lactate with a large T_2_/T_1_ ratio and long T_2_ relaxation times showed a large signal enhancement, whereas glucose and glutamic acid showed enhancements similar to in vivo levels for both CSI‐PC‐bSSFP and ME‐PC‐bSSFP, in comparison to the standard CSI (compare Figure 4 and Figure S5). The large SDs in the estimated values within the small ROI are because of partial volume effects. Although the simulation predicted the general trend of the observed SNR in both phantom and in vivo experiments, the simulated SNR improvements were consistently overestimated. This is expected, because the simulation did not account for SNR degradation factors such as noise amplification during spectral separation,^28^ the effect of the frequency response of relatively long pulses on metabolites with large off‐resonance, the PSF shape because of sampling, uncertainties in relaxation times, and transmit inhomogeneities.

### 4.1 Encoding schemes

In general, CSI encoding is optimal for achieving better spectral encoding, whereas the multi‐echo encoding is optimal for achieving higher spatial resolution. Because of the poor sensitivity of DMI and the sparse ^2^H spectrum, both encoding schemes fulfill the low spectral–spatial resolution requirements of DMI. CSI encoding is typically the preferred method because it offers a higher ADC duty cycle because of less gradient switching, silent operation, and better interpretable spatially resolved frequency spectra. The fast acquisition of multi‐echo encoding could be advantageous in case signal averaging is used for measuring more phase cycles as investigated here.

### 4.2 Phase cycling

The combination of a relatively long TR of 19 ms, required achieving sufficient spectral dispersion, and the significant B_0_ inhomogeneity in the human head at 9.4 T (3.5 ppm≈210 Hz) necessitated the use of phase‐cycling to minimize off‐resonance sensitivity. The high number of 18 phase cycles in ME‐PC‐bSSFP mitigated aliasing in higher order modes and provided additional spectral encoding. As some averages are required for acquisition weighting, only four phase cycles were used in the CSI‐PC‐bSSFP protocol. Because the amplitudes of the higher order SSFP modes decay fast because of the short T_2_ relaxation times relative to TR and the used large flip angles,^22^ no visible aliasing of higher order modes was observed in vivo even with only four phase cycles.

The use of the frequency‐dependent amplitude and phase along phase cycles for spectral separation has been reported previously.^17^, ^44^ Although all protocols in this study were designed to resolve all four metabolites without phase‐cycling, the additional spectral encoding information from phase‐cycling could be used to reduce the TR for increased off‐resonance insensitivity.

The main drawback of using phase‐cycling is essentially the SNR loss because of the low signal in the bSSFP stopband, which is substantially lower than the FISP steady‐state signal (see Figure S8). The SNR loss because of phase‐cycling depends on the metabolite and acquisition parameters. We obtained a decrease of SNR of up to 27% in simulations and of 10% to 20% in subject measurements because of phase‐cycling (see Figure 6). Otherwise, the time penalty of phase‐cycling is negligible because signal averaging already requires multiple repetitions and the transition period between phase cycles is short.

### 4.3 Spectral separation

In addition to improving the signal levels through bSSFP acquisition, it is essential to minimize noise amplification during the spectral separation to retain the SNR advantage. This can be achieved by selecting an appropriate signal model that accurately explains the acquired signal with optimal bias‐variance trade‐off ^45^ and by ensuring proper conditioning during the signal model inversion. A signal model that incorporates phase‐cycling, relaxation times, and B_0_ off‐resonance in addition to the chemical shifts can describe the acquired signal accurately. To ensure proper conditioning, the echo spacing of the multi‐echo protocols and readout lengths of the CSI protocols are chosen to maximize the number of signal averages^23^ (Fisher information) for all ^2^H resonances (see Figure S1).

The IDEAL algorithm used for the standard CSI data fits the signal model by considering only chemical shifts and B_0_ off‐resonance. In case of phase‐cycled bSSFP data, the more descriptive model of the data used in the proposed linear fitting method achieves higher SNR than simply averaging the phase cycles (see Figure 6). As this model includes the amplitude and phase variations because of phase‐cycling, it has the added benefit of providing additional spectral encoding information as shown in Figure 3. However, because of the uncertainty in the estimation of the relaxation times and flip angles, the IDEAL‐modes algorithm using a flexible signal model, which considers only the phase evolution of the metabolites across SSFP configurations, is proposed in this work. It further improves SNR in comparison to the linear fitting method by reducing any bias in the signal model through implicit determination of the relaxation times and flip angles from the data. The optimal weights calculated with Eigen decomposition for combining metabolite amplitudes across SSFP modes show close resemblance to the simulated SSFP mode decay of individual metabolites (see Figure S9). As only the chemical shift and B_0_ off‐resonances are fitted to individual modes, the spectral information in the phase cycles is not used in this method. Moreover, the flexibility of the simple model makes it suitable for application to other field strengths or different tissue types (e.g., tumors) with unknown relaxation times or chemical species with relaxation time heterogeneity such as water in brain tissue and ventricles.

### 4.4 Metabolite quantification

The obtained quantitative metabolite maps were of high quality, without any major artifacts, indicating sufficient SNR throughout the entire brain, even at relatively small voxel sizes. To maximize the data quality for reliable SNR comparison, the acquisitions were performed 30 min after glucose intake. Therefore, the assumed 10.12 mM water reference is in fact slightly higher and results in a minor underestimation of glucose and Glx concentrations. The strong agreement of metabolite concentrations between different methods suggests accurate calibration factors calculated using the respective signal model assumptions of the different acquisition techniques. The ME‐PC‐bSSFP metabolite map demonstrated higher dispersion of metabolite concentrations, especially for Glx, indicating higher resolution and reduced partial volume dilution effects compared to other CSI protocols.

The significant variability in the glucose time course across different subjects necessitates further dynamic DMI investigations with continuous glucose measurement.^42^ All sequences achieved good SNR for glucose allowing the detection of increased glucose uptake in the skull in addition to the brain, as shown in a recent high‐resolution FDG‐PET study.^46^ The raw glucose maps from the ME‐PC‐bSSFP acquisition showed highly localized uptake in the skull, as evidenced in Figure 5 and Figure S10. In some subjects, the stronger glucose signal in the skull is because of a combination of late measurement time, receiver sensitivity bias, and rapid metabolic conversion of glucose in the brain. However, the quantification of this glucose signal is challenging because of the absence of a reliable water reference in the skull.

### 4.5 System consideration

The reference power was set lower because of the limited RF peak power of approximately 4 kW (447 V peak amplitude) and the vendor's convention of defining reference power with a 0.5 ms RF pulse duration. As a result, the actual flip angles achieved were 85% to 90% of the nominal flip angles. The limited power availability also led to longer inversion and refocusing pulses in the relaxometry experiments. A small variation in B_1_^+^ efficiency of 147 to 160 nT/V, calculated from the reference amplitude measurements, was observed across different head sizes. Given the simulated maximum local SAR (SAR_10g_) of 0.296 W/kg for 1 W input power, safety margins because of simulation inaccuracies, and increased measurement uncertainty of directional couplers approximately 61 MHz, the allowed maximum instantaneous time‐averaged power was approximately 12 W for all sequences lasting more than 6 min. Therefore, the pulse duration of the bSSFP acquisitions was increased to 1.4 ms to achieve high flip angles. On the receiver side, adding a second‐stage amplification of 18 dB (high gain option) to the 26 dB power gain of the pre‐amplifier significantly improved SNR, indicating a dominant noise contribution from the receive chain.

## 5 CONCLUSION

This work successfully developed two robust phase‐cycled bSSFP acquisition and novel processing methods for improving DMI at a human 9.4 T scanner for investigating the healthy human brain. All experiments were performed at an improved spatial resolution of 2.7 to 4.3 mL (equivalent of acquisition‐weighted 0.4–0.6 mL nominal resolution) and high temporal resolution of 10 min. bSSFP acquisition has potential to improve the sensitivity of DMI despite the SNR loss of phase‐cycling and other human scanner safety constraints.

## FUNDING INFORMATION

Financial support from the Max‐Planck‐Society, Deutsche Forschungsgemeinschaft (the Reinhart Koselleck Project) (SCHE658/S2), the European Research Council (Spread MRI) (Grant 834940), and Bundesministerium für Bildung und Forschung (01GQ1805B) is gratefully acknowledged. We also acknowledge the support from the Deutsche Forschungsgemeinschaft (DFG, German Research Foundation) by the Cluster of Excellence iFIT (EXC 2180) “Image‐Guided and Functionally Instructed Tumor Therapies,” University of Tuebingen, Germany (KS and AFM), the Werner Siemens Foundation, the Alexander von Humboldt Foundation within the framework of the Sofja Kovalevskaja Award (AFM), and the DKTK German Cancer Consortium Innovation program "HYPERBOLIC“ (LK).

## Supporting information


**Table S1.** Overview of the spatial resolution, acquisition time and other important acquisition parameters of all published human deuterium metabolic imaging studies. All the studies listed above except the ME‐bSSFP protocol of this study use acquisition weighting to improve the sensitivity of DMI at the expense of spatial resolution.
**Figure S1.** The normalized number of signal averages (NSA) or the Fisher information estimated from the system model matrix with four ^2^H resonances at different echo spacings for the multi‐echo acquisition and different readout lengths for the CSI acquisitions. The magenta and cyan line markers indicate the echo‐spacing and readout lengths used in the optimized protocols of this study.
**Figure S2.** A detailed description of the proposed IDEAL‐modes algorithm for metabolite amplitude estimation. The inputs, outputs and key computational steps involved after image reconstruction are outlined.
**Figure S3.** Non‐localized inversion recovery T_1_ measurements performed approximately 90 minutes after glucose intake in subject 1. The top row shows the inversion recovery data, the corresponding 2D fit, and the residuals. The bottom row presents the fit across all inversion times, along with the measured data points and residuals. Residuals are vertically offset by 0.2 for clarity.
**Figure S4.** Non‐localized spin‐echo T_2_ measurements performed approximately 100 minutes after glucose intake in subject 1. The top row shows the spin‐echo data, the corresponding 2D fit, and the residuals. The bottom row presents the fit across all echo times, along with the measured data points and residuals. Residuals are vertically offset by 0.1 for clarity.
**Figure S5.** Simulated phantom SNR efficiency ($\text{signal amplitude}/\sqrt{\mathrm{TR}}$) of deuterium metabolites as a function of TR and flip angle for standard CSI, CSI‐PC‐bSSFP and ME‐PC‐bSSFP acquisitions. The measured phantom relaxation times used for the simulation are shown in the title of the respective subplots. The SAR limits with respect to TR for three pulse durations are overlaid to show the available parameter space for SNR optimization. The asterisks in the plots indicate the parameter combination (flip angle, TR) of the protocols used in this study. The signal efficiencies of all the three protocols for all four metabolites are shown in the bottom panel. The relative improvement over the standard CSI is shown on top of the bar plots.
**Figure S6.** The Hamming weighting of the two CSI protocols and the elliptical sampling of ME‐PC‐bSSFP are shown on the top row. The corresponding point spread function (PSF) calculated from the k‐space weights are depicted on the bottom panels along with their full width at 64% (FW64%) in mm. In the case of CSI protocols with 8.3 mm nominal isotropic resolution, isotropic hamming weightings and PSFs are shown for one direction. For ME‐PC‐bSSFP with 12.5 mm nominal isotropic resolution, the PSF is different along the read and phase encoding directions. The CSI‐PC‐bSSFP has a larger PSF voxel size compared to the standard CSI because of the discretization error due to the reduced number of averages.
**Figure S7.** SNR improvements for all metabolites with the protocol parameters (TR, flip angle, and duty cycle) and relaxation times at the respective field strength using the signal model used in this work. The model predicts higher SNR improvements for bSSFP as reported in the corresponding publications.
**Figure S8.** The in vivo bSSFP signal level (solid line) of three metabolites for different RF phase increments is shown on the left along with the FISP signal level (dashed line). The signal levels are simulated for the protocols used in this study and the measured in vivo relaxation times at 9.4 T. Similarly, the bSSFP signal level is shown on the right along with the FISP signal level for all four metabolites with the relaxation times measured in the phantom.
**Figure S9.** The SSFP mode amplitudes estimated from the simulated bSSFP frequency response for the measured in vivo relaxation times and a 50° flip angle. The principal eigenvectors of all three metabolites estimated from the fitted metabolite amplitudes over different modes.
**Figure S10.** Evidence of glucose signal in the human skull. (A) Axial slices of ME‐bSSFP glucose SNR maps for all subjects with anatomical underlay showing highly resolved glucose uptake in the skull and eyes in addition to the brain. The acquisition time after the glucose intake is shown for each subject. The maps are not corrected for receive sensitivity. (B) Field maps were estimated by the IDEAL algorithm from the same dataset for all subjects. The spectra from skull and brain ROIs from a pilot CSI dataset with higher spectral resolution are presented for two slices in C and D, respectively. The DMI glucose signal in the human skull was previously reported by Ruhm et al. 2021^9^ (see Figure 10 therein).

## Data Availability

The MATLAB software package used for simulation and DMI processing are available on Github at https://github.com/praveenivp/DeuteMetcon.git (v0.2.0). The Pulseq sequences and associated processing scripts for estimating T_1_, T_2_, and reference voltage were also shared in the same repository. The DMI raw data from both phantom and subjects as well as non‐localized spectroscopy data is openly available in zenodo: https://doi.org/10.5281/zenodo.15864245. The instruction manual for phantom preparation and the measurement protocols are also shared along with the data repository. Sequence binaries can be obtained through the Siemens Healthineers customer‐to‐customer partnership program (C2P procedure) on reasonable request.
