# Supplementary material for: High‐resolution deuterium metabolic imaging of the human brain at 9.4 T using phase‐cycled balanced SSFP spectral–spatial acquisitions
Source: Magn Reson Med. 2025 Oct 13;95(3):1304–22. doi: 10.1002/mrm.70114 (PMC12746357; doi:10.1002/mrm.70114)
Supplement: Supplementary file 1 — Table S1. Overview of the spatial resolution, acquisition time and other important acquisition parameters of all published human deuterium metabolic imaging studies. All the studies listed above except the ME‐bSSFP protocol of this study use acquisition weighting to improve the sensitivity of DMI at the expense of spatial resolution. Figure S1. The normalized number of signal averages (NSA) or the Fisher information estimated from the system model matrix with four 2H resonances at different echo spacings for the multi‐echo acquisition and different readout lengths for the CSI acquisitions. The magenta and cyan line markers indicate the echo‐spacing and readout lengths used in the optimized protocols of this study. Figure S2. A detailed description of the proposed IDEAL‐modes algorithm for metabolite amplitude estimation. The inputs, outputs and key computational steps involved after image reconstruction are outlined. Figure S3. Non‐localized inversion recovery T1 measurements performed approximately 90 minutes after glucose intake in subject 1. The top row shows the inversion recovery data, the corresponding 2D fit, and the residuals. The bottom row presents the fit across all inversion times, along with the measured data points and residuals. Residuals are vertically offset by 0.2 for clarity. Figure S4. Non‐localized spin‐echo T2 measurements performed approximately 100 minutes after glucose intake in subject 1. The top row shows the spin‐echo data, the corresponding 2D fit, and the residuals. The bottom row presents the fit across all echo times, along with the measured data points and residuals. Residuals are vertically offset by 0.1 for clarity. Figure S5. Simulated phantom SNR efficiency (signal amplitude/TR) of deuterium metabolites as a function of TR and flip angle for standard CSI, CSI‐PC‐bSSFP and ME‐PC‐bSSFP acquisitions. The measured phantom relaxation times used for the simulation are shown in the title of the respective subplots. The SAR limits with r [file MRM-95-1304-s001.docx]

Supporting Information

# High-resolution deuterium metabolic imaging of the human brain at 9.4 T using phase-cycled bSSFP spectral-spatial acquisitions

Praveen Iyyappan Valsala^1,2^, Rolf Pohmann^1^, Rahel Heule^1,2,3^, Georgiy A. Solomakha^1^, Nikolai Avdievich^1^, Jörn Engelmann^1^, Laura Kuebler^4,5^, André F. Martins^4,5^ , Klaus Scheffler^1,2^

*^1^High Field Magnetic Resonance, Max-Planck Institute for Biological Cybernetics, Tübingen, Germany*

*^2^Department of Biomedical Magnetic Resonance, Eberhard Karls University Tübingen, Tübingen, Germany*

*^3^Center for MR Research, University Children's Hospital, Zurich, Switzerland*

*^4^Werner Siemens Imaging Center, Department for Preclinical Imaging and Radiopharmacy, Eberhard Karls University Tübingen*

*^5^Cluster of Excellence iFIT (EXC 2180) «Image-Guided and Functionally Instructed Tumor Therapies», Eberhard Karls University Tübingen*

| Studies | Nominal Resolution  [mm] | Acquisition  Type | Acquisition time  [min] | Field Strength  [T] | TR  [ms] | FA  [deg] | DC  [%] |
| --- | --- | --- | --- | --- | --- | --- | --- |
| [De Feyter et al. 2018](https://doi.org/10.1126/sciadv.aat7314) | 20x20x20  (8 mL) | 3D CSI | 29 | 4 | 333 | 90 | n.a |
| [Ruhm et al. 2021](https://doi.org/10.1016/j.neuroimage.2021.118639) | 15x15.3x12.8  (3 mL) | 3D CSI | 10 | 9.4 | 155 | 51 | 66 |
| [Roig et al. 2022](https://doi.org/10.1002/mrm.29439) | 14x14x14  (2.7 mL) | 3D CSI | 28 | 7 | 350 | 90 | 29 |
| [Kaggie et al. 2022](https://doi.org/10.1016/j.neuroimage.2022.119284) | 32x32x32  (33 mL) | 3D CSI | 10 | 3 | 120 | 90 | 90.6 |
| [Adamson et al. 2023](https://doi.org/10.1002/mrm.29830) | 24x24x24  (13.8 mL) | 3D CSI | 39 | 3 | 350 | 90 | 73.1 |
| [Niess et al. 2024](https://doi.org/10.1002/hbm.26686) | 12.5x12.5x12.5  (2 mL) | 3D CSI | 7 | 7 | 290 | 86 | 87 |
|  | 9.1x9.1x8.3  (0.75 mL) | 3D CRT | 7 | 7 | 290 | 86 | 87 |
| [Frese et al. 2025](https://doi.org/10.1101/2025.02.06.25321580)  (preprint) | 7.1x7.1x7.1  (0.36 mL) | 3D CRT | 7 | 7 | 290 | 86 | 87 |
|  | 7.1x7.1x7.1  (0.36 mL) | 3D CRT-bSSFP | 7 | 7 | 23 | 50 | 76 |
| **This study** | **8.3 x8.3x8.3**  **(0.58 mL)** | **3D CSI** | **10** | **9.4** | **36** | **41** | **79** |
|  | **8.3 x8.3x8.3**  **(0.58 mL)** | **3D CSI-PC-bSSFP** | **10** | **9.4** | **19** | **50** | **78** |
|  | **12.5x12.5x12.5**  **(1.95 mL)** | **3D ME-PC-bSSFP** | **10** | **9.4** | **19** | **50** | **71** |

**Supporting Information Table ST1:** Overview of the spatial resolution, acquisition time and other important acquisition parameters of all published human deuterium metabolic imaging studies. All the studies listed above except the ME-PC-bSSFP protocol of this study use acquisition weighting to improve the sensitivity of DMI at the expense of spatial resolution. (TR, repetition time; FA , flip angle ; CRT, concentric ring trajectory; CSI, chemical shift imaging; ME, multi-echo; bSSFP, balanced steady state free precession; DC, ADC duty cycle)


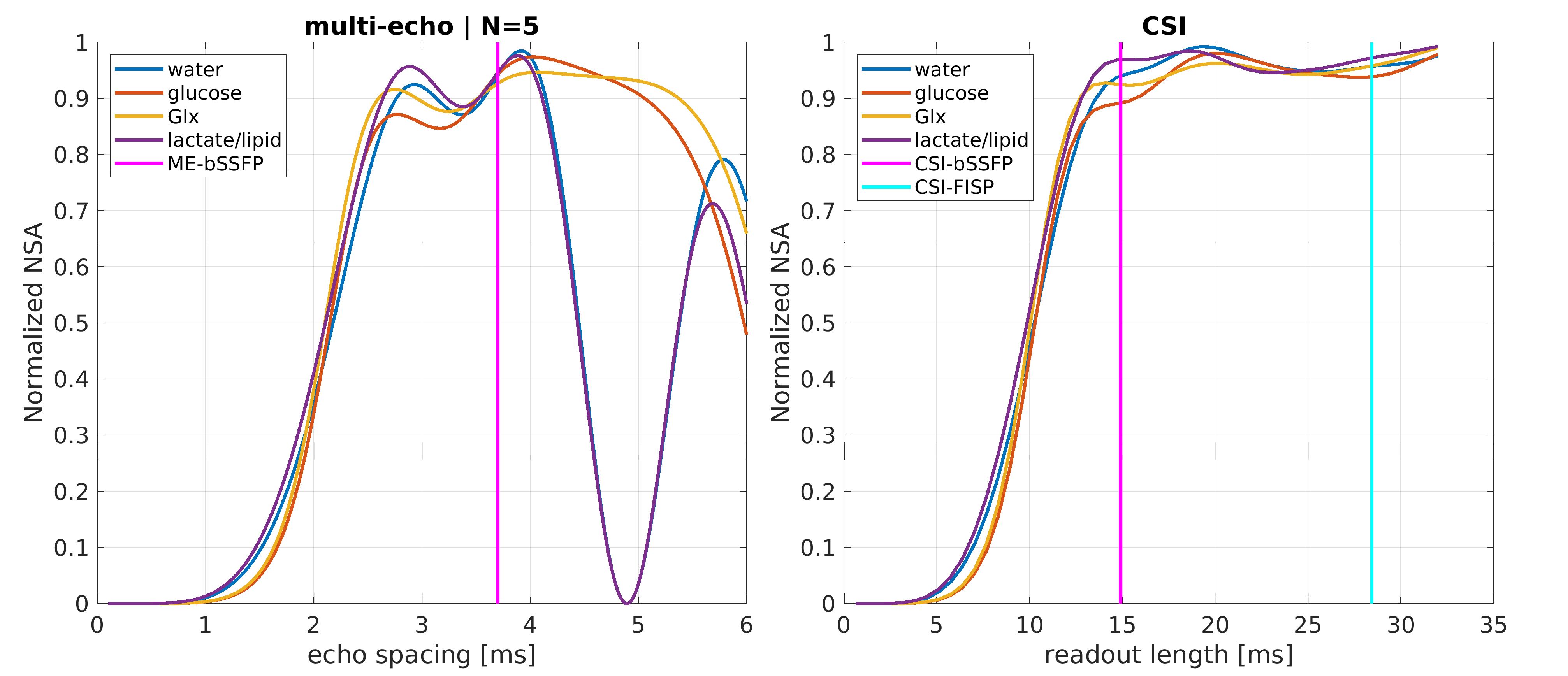


**Supporting Information Figure S1:** The normalized number of signal averages (NSA) or the Fisher information estimated from the system model matrix with four ^2^H resonances at different echo spacings for the multi-echo acquisition and different readout lengths for the CSI acquisitions. The magenta and cyan line markers indicate the echo-spacing and readout lengths used in the optimized protocols of this study.

**Supporting Information Figure S2**: A detailed description of the proposed IDEAL-modes algorithm for metabolite amplitude estimation. The inputs, outputs and key computational steps involved after image reconstruction are outlined.

**Algorithm for IDEAL-Modes method**

**Inputs**:

- K phase-cycled bSSFP volumes $I(k,t_{n})$ at $N$ time points $t_{n}=\{t_{1},t_{2},\ldots,t_{N}\}$.
- Chemical shifts $\Delta f_{j}$​ for $M$ metabolites.
- Repetition time ($\text{TR}$)
- RF phase increment in radians $\psi_{k}$ for K phase cycles

**Output**:

- Metabolite amplitude maps $\rho_{j}$ for $M$ metabolites.

**Procedure:**

**1. Calculate SSFP Configuration Modes with order c**

$F\left( c,t_{n} \right)=\sum_{k=0}^{K} I\left( k,t_{n} \right)e^{i\psi_{k}c} \forall c=-C,\ldots,C$, where $C= \left\lfloor\frac{K-1}{2} \right\rfloor$ [A1]

**2. Estimate Off-Resonance map** $\boldsymbol{\Delta}\boldsymbol{f}_{\boldsymbol{0}}$ **with IDEAL algorithm with** $F(0,t_{n})$

**3. Calculate and store all metabolite amplitudes across modes**

**for each voxels:**

Calculate phase terms defined in the methods

$\varphi_{j}\left( t_{n} \right):=\left( {t_{n}}/{TR} \right)\Phi_{j}$ and $\Phi_{j}:=2\pi\left( \Delta f_{j}+\Delta f_{0} \right)\mathrm{TR}$ [A2]

**for each mode** $c=-C,\ldots,C$

Construct design matrix $A_{c}$ and data column vector $b_{c}$ to estimate mode amplitudes $b_{c}$

$A_{c}=\left[ \begin{matrix} e^{i\varphi_{1}(t_{1})}e^{ic\Phi_{1}} & e^{i\varphi_{2}(t_{1})}e^{ic\Phi_{2}} & \cdots& e^{i\varphi_{M}(t_{1})}e^{ic\Phi_{M}} \\ e^{i\varphi_{1}(t_{2})}e^{ic\Phi_{1}} & e^{i\varphi_{2}(t_{2})}e^{ic\Phi_{2}} & \ldots& e^{i\varphi_{M}(t_{2})}e^{ic\Phi_{M}} \\ \vdots& \vdots& \ddots& \vdots\\ e^{i\varphi_{1}(t_{N})}e^{ic\Phi_{1}} & e^{i\varphi_{2}(t_{N})}e^{ic\Phi_{2}} & \cdots& e^{i\varphi_{M}(t_{N})}e^{ic\Phi_{M}} \end{matrix} \right]$, $x_{c}=\left[ \begin{matrix} P_{1,c} \\ P_{2,c} \\ \vdots\\ P_{M,c} \end{matrix} \right]$,$b_{c}=\left[ \begin{matrix} F(c,t_{1}) \\ F(c,t_{2}) \\ \vdots\\ F(c,t_{N}) \end{matrix} \right]$ [A3]

Solve the linear system of equation ${A_{c}x}_{c}=b_{c}$and estimate metabolite amplitude for each mode:

$\hat{x_{c}}={{(A}_{c}^{H}A_{c})}^{-1}A_{c}^{H} b_{c}$ [A4]

**end**

Assemble all metabolite amplitudes in all SSFP modes:

$\hat{P}_{j,c}=\left[ \begin{matrix} x_{-C} & \ldots& x_{C} \end{matrix} \right]=\left[ \begin{matrix} \hat{P}_{1,-C} & \ldots& \hat{P}_{1,0} & \ldots& \hat{P}_{1,C} \\ \hat{P}_{2,-C} & \ldots& \hat{P}_{2,0} & \ldots& \hat{P}_{2,C} \\ \vdots& \ddots& \vdots& \ddots& \vdots\\ \hat{P}_{M,-C} & \cdots& \hat{P}_{M,0} & \cdots& \hat{P}_{M,C} \end{matrix} \right]$ **[A5]**

**end**

**4. Optimally combine metabolite amplitudes across modes to obtain final metabolite map** $\boldsymbol{\rho}_{\boldsymbol{j}}$ **for j^th^ metabolite in all voxels**

Perform SVD decomposition of $\hat{B}_{j}$

$\hat{B}_{j}=\left[ \begin{matrix} \left( \begin{matrix} \hat{P}_{j,-C} & \ldots& \hat{P}_{j,C} \end{matrix} \right)_{voxel 1} \\ \left( \begin{matrix} \hat{P}_{j,-C} & \ldots& \hat{P}_{j,C} \end{matrix} \right)_{voxel 2} \\ \left( \begin{matrix} \hat{P}_{j,-C} & \ldots& \hat{P}_{j,C} \end{matrix} \right)_{voxel 3} \\ \vdots\end{matrix} \right]=U_{j}\Sigma_{j}V_{j}^{H}$ **[A6]**

Extract the first principal component $c_{j}={{(V}_{j})}_{:,1}$ and combined metabolite amplitudes across modes

$\hat{\rho}_{j}=\hat{B}_{j}c_{j}$ **[A7]**


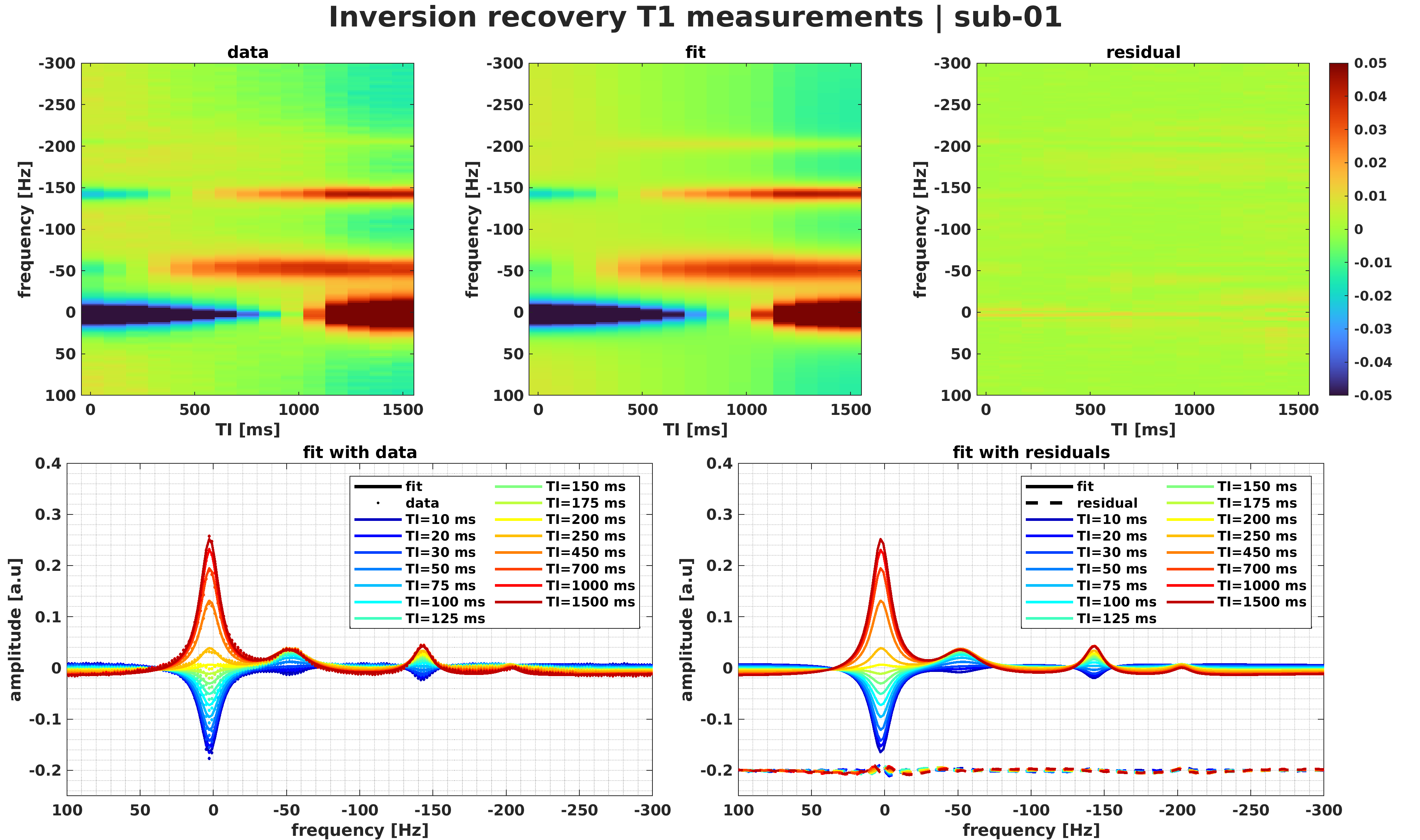


**Supporting Information Figure S3:** Non-localized inversion recovery T_1_ measurements performed approximately 90 minutes after glucose intake in subject 1. The top row shows the inversion recovery data, the corresponding 2D fit, and the residuals. The bottom row presents the fit across all inversion times, along with the measured data points and residuals. Residuals are vertically offset by 0.2 for clarity.


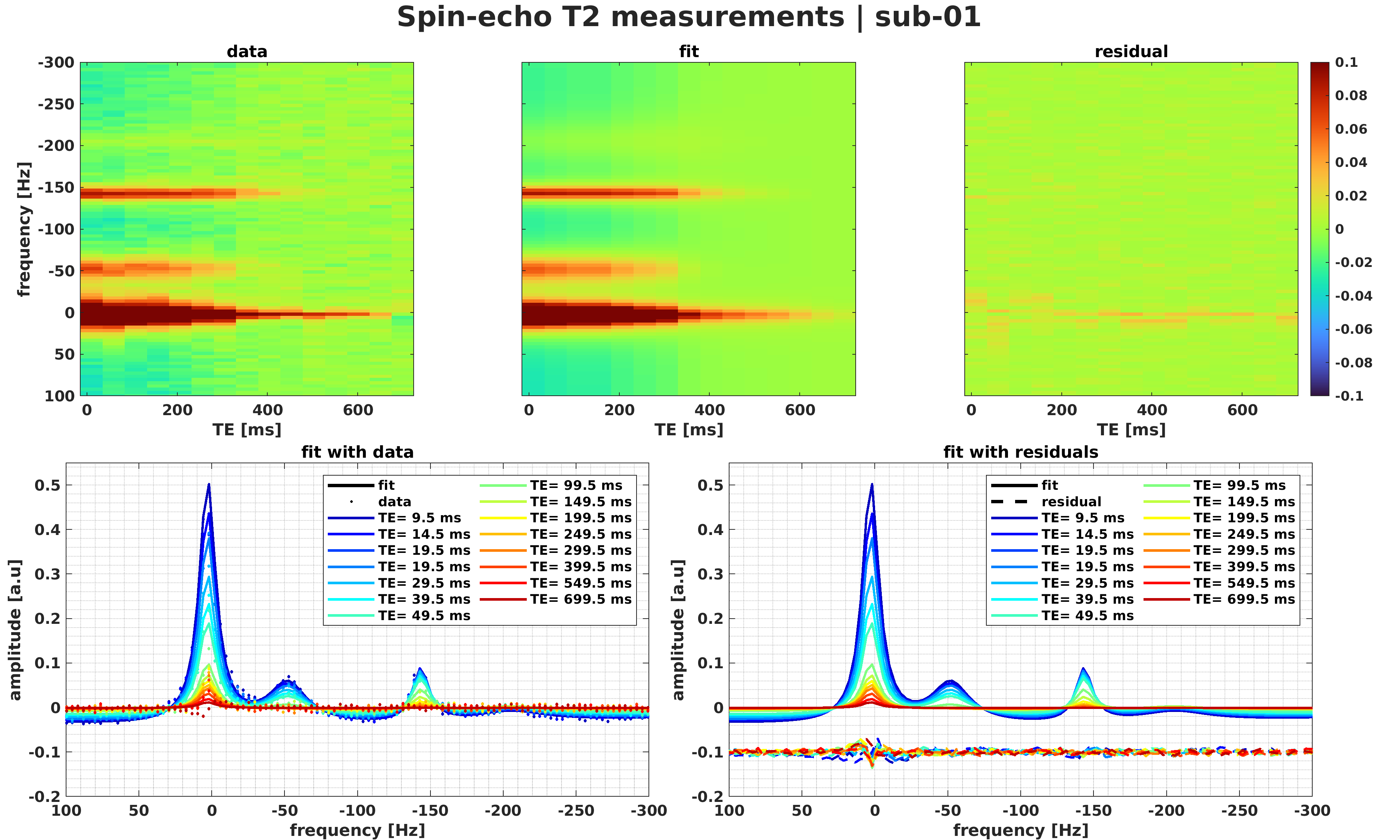


**Supporting Information Figure S4:** Non-localized spin-echo T_2_ measurements performed approximately 100 minutes after glucose intake in subject 1. The top row shows the spin-echo data, the corresponding 2D fit, and the residuals. The bottom row presents the fit across all echo times, along with the measured data points and residuals. Residuals are vertically offset by 0.1 for clarity.


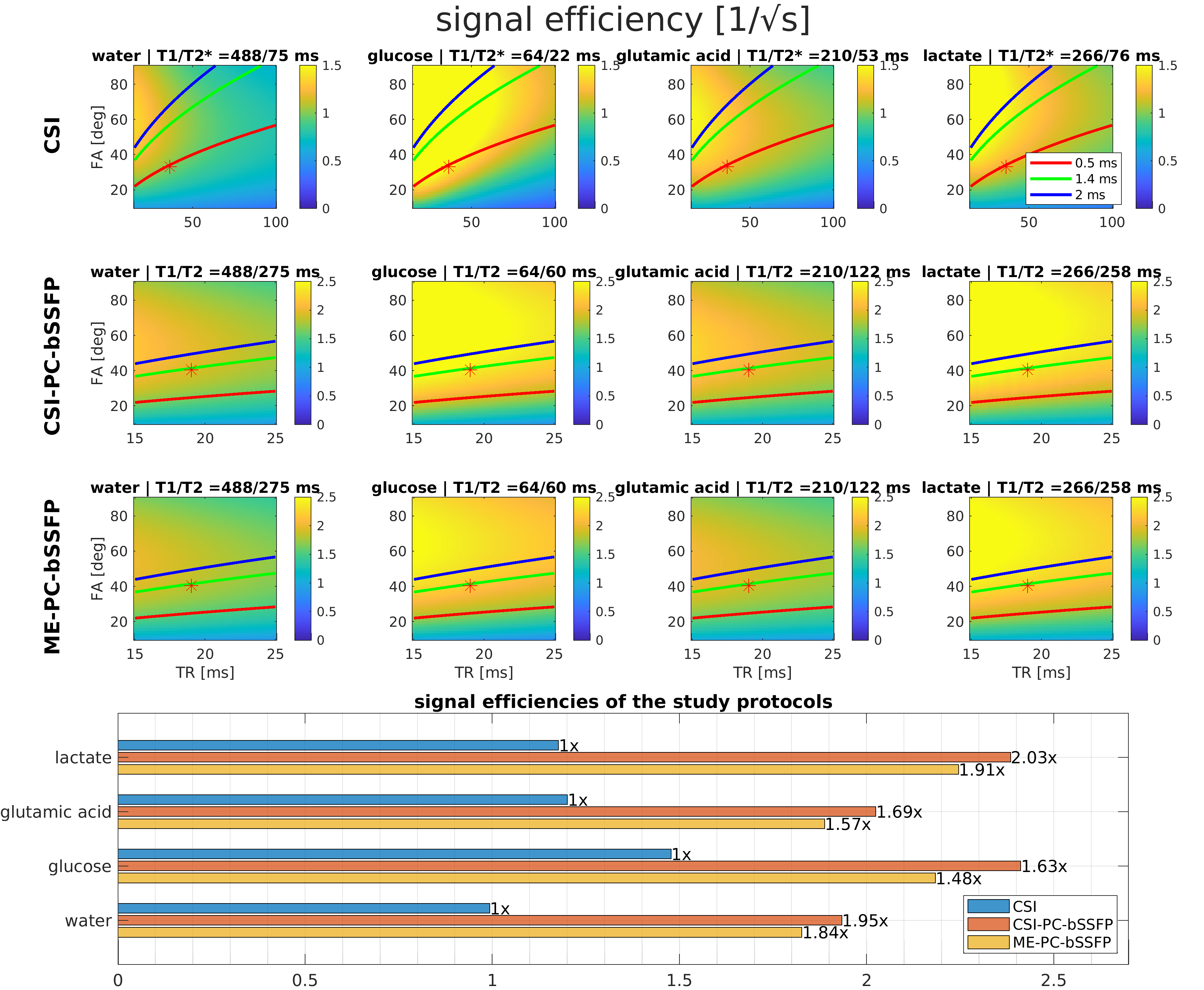


**Supporting Information Figure S5:** Simulated phantom SNR efficiency (signal amplitude/$\sqrt{TR}$) of deuterium metabolites as a function of TR and flip angle for standard CSI, CSI-PC-bSSFP and ME-PC-bSSFP acquisitions. The measured phantom relaxation times used for the simulation are shown in the title of the respective subplots. The SAR limits with respect to TR for three pulse durations are overlaid to show the available parameter space for SNR optimization. The asterisks in the plots indicate the parameter combination (flip angle, TR) of the protocols used in this study. The signal efficiencies of all the three protocols for all four metabolites are shown in the bottom panel. The relative improvement over the standard CSI is shown on top of the bar plots.


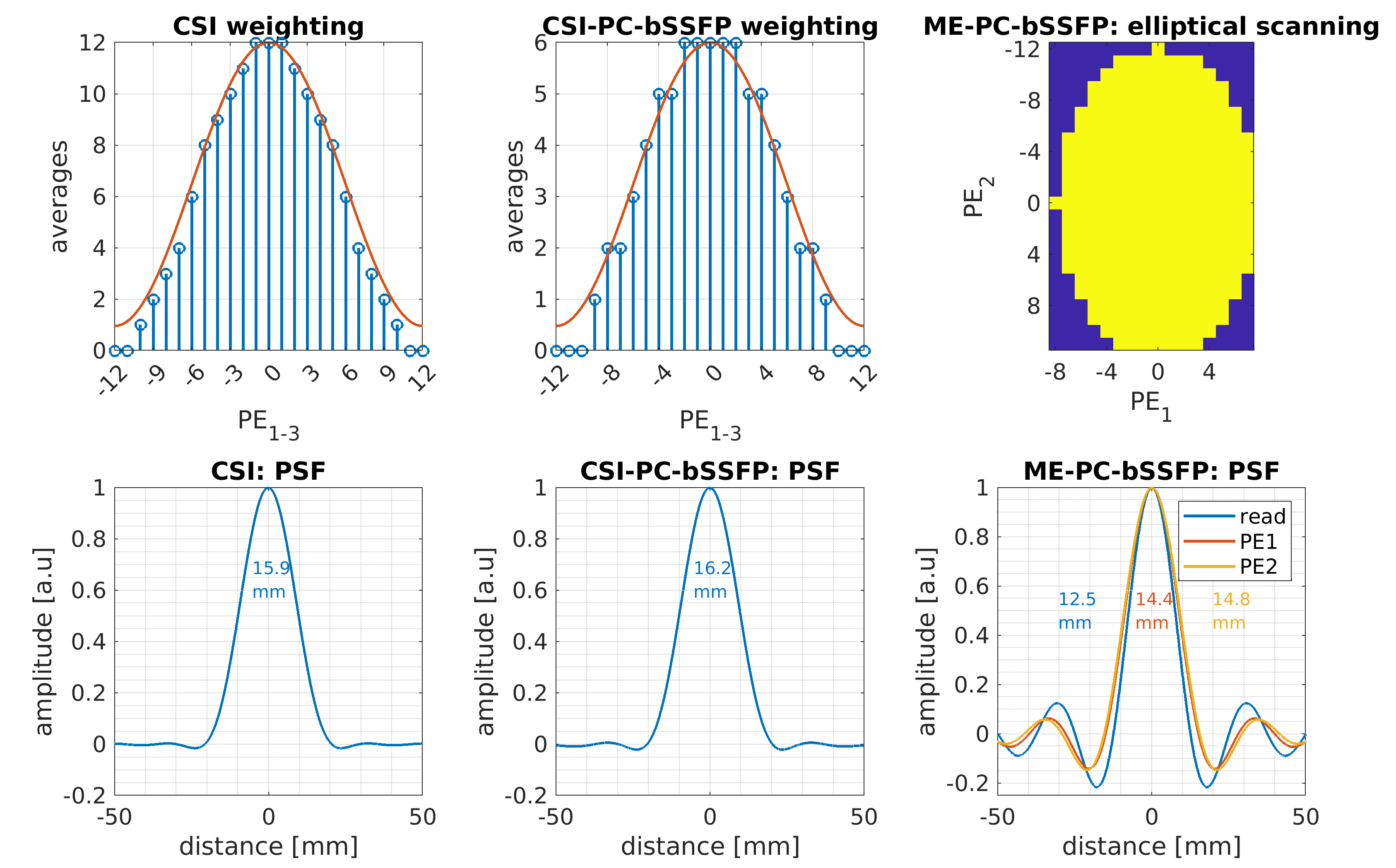


**Supporting Information Figure S6:** The Hamming weighting of the two CSI protocols and the elliptical sampling of ME-PC-bSSFP are shown on the top row. The corresponding point spread functions (PSF) calculated from the k-space weights are depicted on the bottom panels along with their full width at 64% (FW64%) in mm. In the case of CSI protocols with 8.3 mm nominal isotropic resolution, isotropic hamming weightings and PSFs are shown for one direction. For ME-PC-bSSFP with 12.5 mm nominal isotropic resolution, the PSF is different along the read and phase encoding directions. The CSI-PC-bSSFP has a larger PSF voxel size compared to the standard CSI because of the discretization error due to the reduced number of averages.


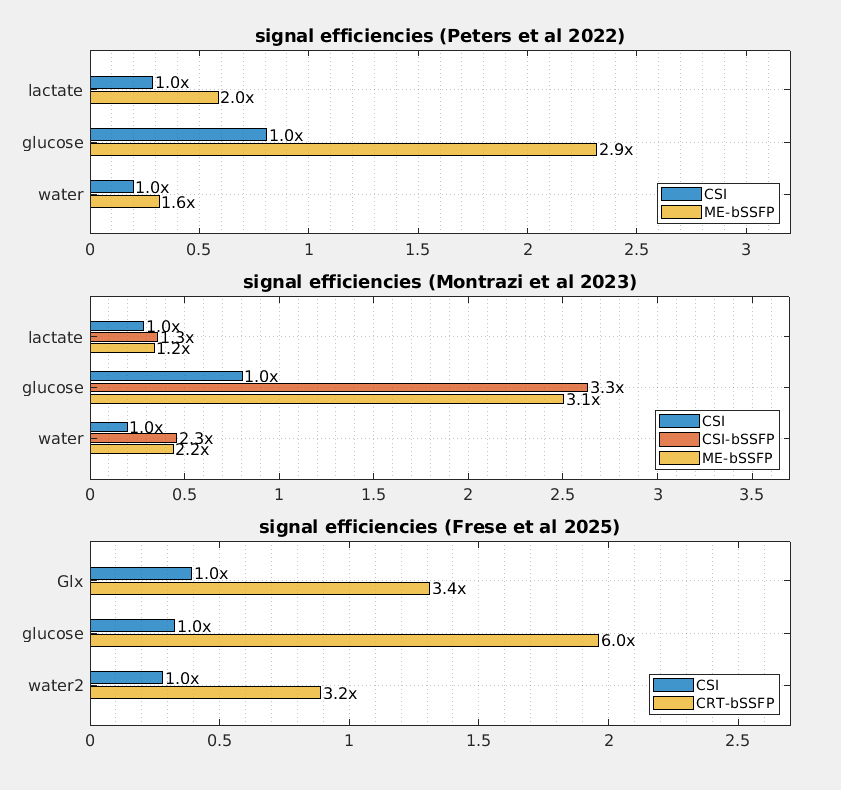


**Supporting Information Figure S7**: SNR improvements for all metabolites with the protocol parameters (TR, flip angle, DC, duty cycle) and relaxation times at the respective field strength. The model predicts higher SNR improvements for bSSFP as reported in the corresponding publications. The reference CSI of both preclinical papers use FLASH signal model from Peters et al. 2022^1^, while the reference CSI used in the human DMI publication uses the FISP signal model^2^. For Peters et al. 2022^1^, DMI protocols (**CSI:** TR=95 ms, DC=84% and FA=90°, **ME-bSSFP:** TR=12.2ms, DC=70% and FA=80°) and relaxation times were obtained from the same paper. For Montrazi et al. 2023^3^, protocols (**CSI:** TR=95 ms, DC=84% and FA=90°, **CSI-bSSFP:** TR=11.48 ms, DC=79% and FA=60°, **ME-bSSFP:** TR=11.48 ms, DC=70% and FA=60°) and relaxation times at 15.2 T from Peters et al. 2022^1^ were used. The human DMI improvements at 7T reported in the recent preprint of Frese et al. 2025^4^ used an even more sub-optimal standard CSI protocol (**CSI:** TR=290 ms, DC=87% and FA=86°, **ME-bSSFP:** TR=23 ms, DC=76% and FA=50°). In vivo relaxation times at 7T were obtained from Roig et al. 2024^5^.


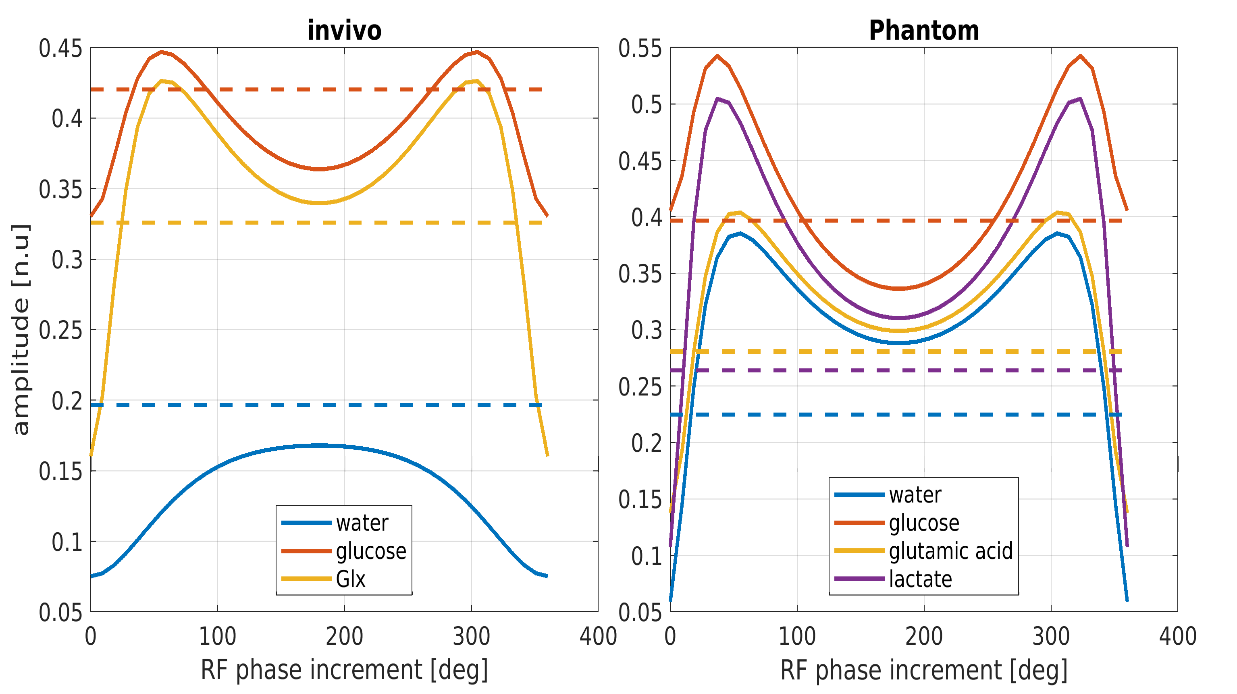


**Supporting Information Figure S8:** The in vivo bSSFP signal level (solid line) of three metabolites for different RF phase increments is shown on the left along with the FISP signal level (dashed line). The signal levels are simulated for the protocols used in this study and the measured in vivo relaxation times at 9.4 T. Similarly, the bSSFP signal level is shown on the right along with the FISP signal level for all four metabolites with the relaxation times measured in the phantom.


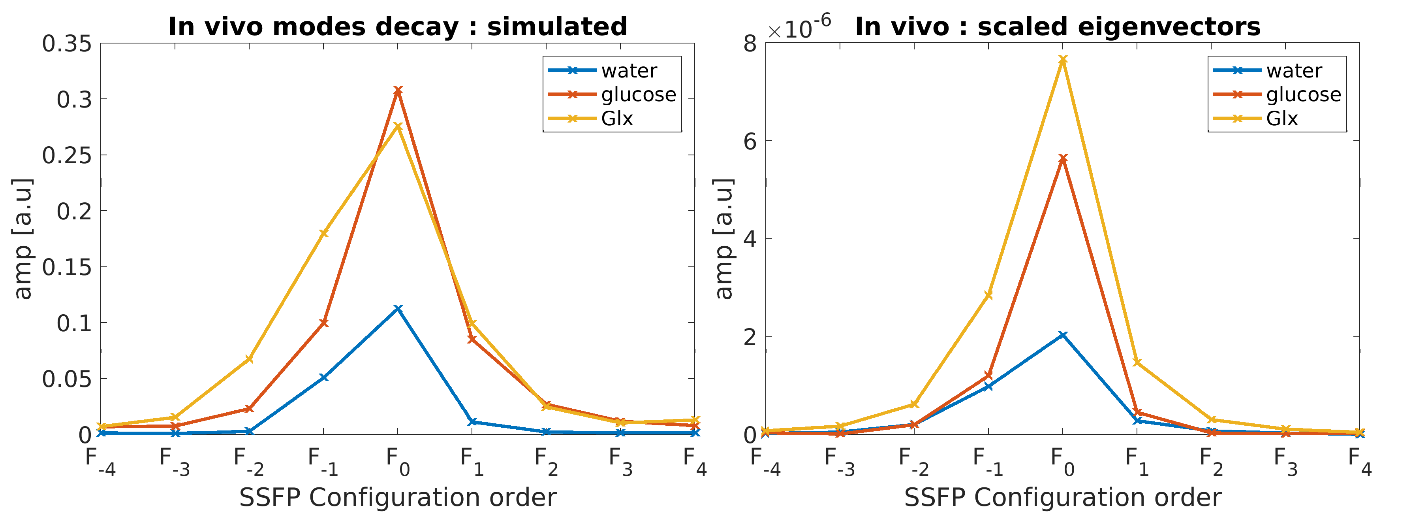


**Supporting Information Figure S9:** The SSFP mode amplitudes estimated from the simulated bSSFP frequency response for the measured in vivo relaxation times and a 50° flip angle. The principal eigenvectors of all three metabolites estimated from the fitted metabolite amplitudes over different modes.


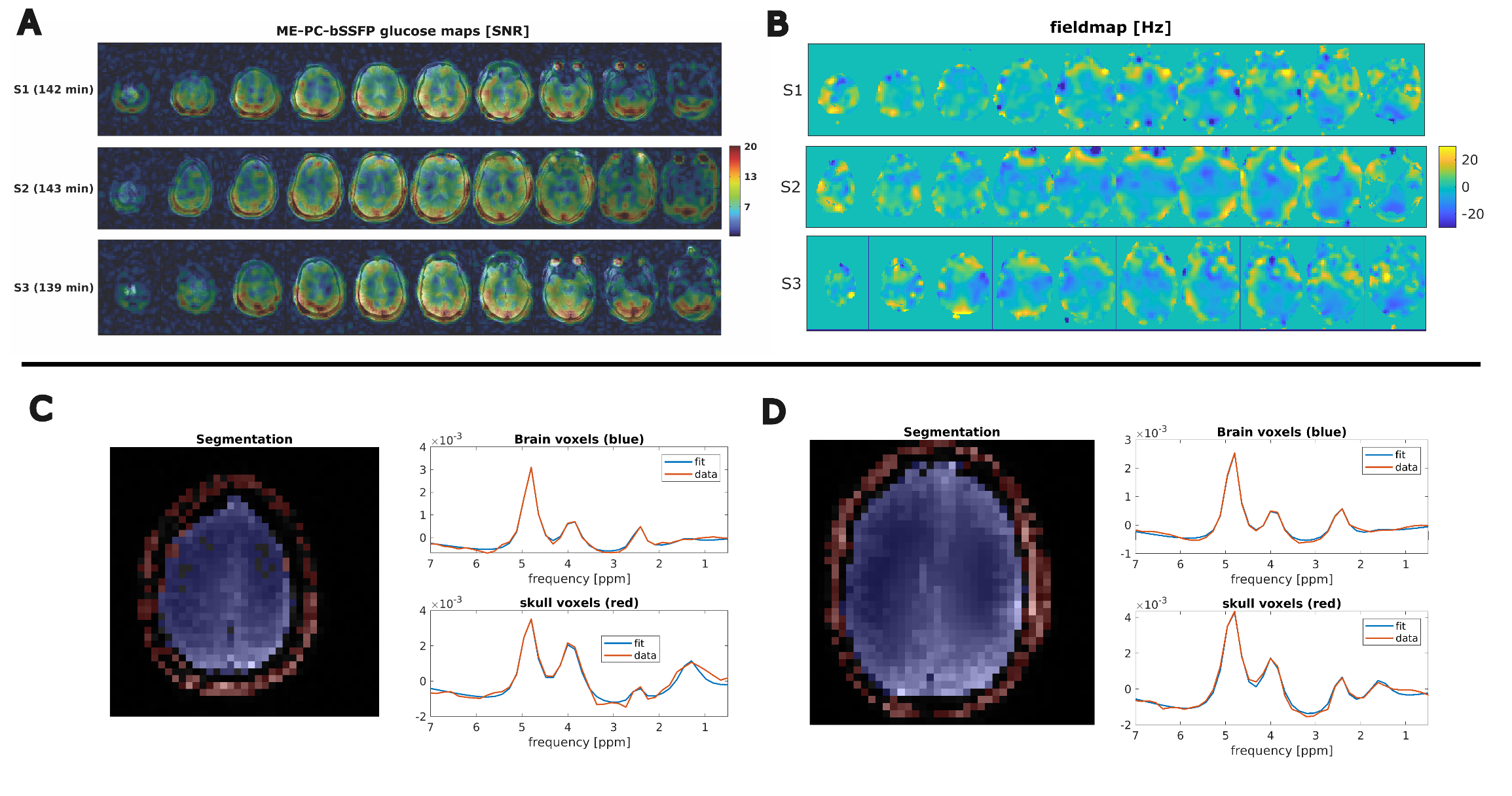


**Supporting Information Figure S10:** Evidence of glucose signal in the human skull. (A) Axial slices of ME-PC-bSSFP glucose SNR maps for all subjects with anatomical underlay showing highly resolved glucose uptake in the skull and eyes in addition to the brain. The acquisition time after the glucose intake is shown for each subject. The maps are not corrected for receive sensitivity. (B) Field maps were estimated by the IDEAL algorithm from the same dataset for all subjects. The spectraspectra from skull and brain ROIs from a pilot CSI dataset with higher spectral resolution are presented for two slices in C and D respectively. The DMI glucose signal in the human skull was previously reported by Ruhm et al. 2021^6^ (see Figure 10 therein)

# References

1. Peters DC, Markovic S, Bao Q, et al. Improving deuterium metabolic imaging (DMI) signal‐to‐noise ratio by spectroscopic multi‐echo bSSFP: A pancreatic cancer investigation. *Magn Reson Med*. 2021;86(5):2604-2617. doi:10.1002/mrm.28906

2. Hänicke W, Vogel HU. An analytical solution for the SSFP signal in MRI. *Magnetic Resonance in Medicine*. 2003;49(4):771-775. doi:10.1002/mrm.10410

3. Montrazi ET, Sasson K, Agemy L, et al. High-sensitivity deuterium metabolic MRI differentiates acute pancreatitis from pancreatic cancers in murine models. *Sci Rep*. 2023;13(1):19998. doi:10.1038/s41598-023-47301-7

4. Frese S, Strasser B, Hingerl L, et al. Balanced steady state free precession enables high-resolution dynamic 3D Deuterium Metabolic Imaging of the human brain at 7T. February 2025. doi:10.1101/2025.02.06.25321580

5. Serés Roig E, De Feyter HM, Nixon TW, et al. Deuterium metabolic imaging of the human brain in vivo at 7 T. *Magnetic Resonance in Medicine*. 2023;89(1):29-39. doi:10.1002/mrm.29439

6. Ruhm L, Avdievich N, Ziegs T, et al. Deuterium metabolic imaging in the human brain at 9.4 Tesla with high spatial and temporal resolution. *NeuroImage*. 2021;244:118639. doi:10.1016/j.neuroimage.2021.118639
